# Underwater image enhancement using Divide-and-Conquer network

**DOI:** 10.1371/journal.pone.0294609

**Published:** 2024-03-05

**Authors:** Shijian Zheng, Rujing Wang, Guo Chen, Zhiliang Huang, Yue Teng, Liusan Wang, Zhigui Liu

**Affiliations:** 1 Department of Information Engineering, Southwest University of Science and Technology, Mianyang, Sichuan, China; 2 Institute of Intelligent Machines, Hefei Institutes of Physical Science, Chinese Academy of Science, Hefei, Anhui, China; 3 Department of Information Engineering, University of Science and Technology of China, Hefei, Anhui, China; University of Engineering & Technology, Taxila, PAKISTAN

## Abstract

Underwater image enhancement has become the requirement for more people to have a better visual experience or to extract information. However, underwater images often suffer from the mixture of color distortion and blurred quality degradation due to the external environment (light attenuation, background noise and the type of water). To solve the above problem, we design a Divide-and-Conquer network (DC-net) for enhancing underwater image, which mainly consists of a texture network, a color network and a refinement network. Specifically, the multi-axis attention block is presented in the texture network, which combine different region/channel features into a single stream structure. And the color network employs an adaptive 3D look-up table method to obtain the color enhanced results. Meanwhile, the refinement network is presented to focus on image features of ground truth. Compared to state-of-the-art (SOTA) underwater image enhance methods, our proposed method can obtain the better visual quality of underwater images and better qualitative and quantitative performance. The code is publicly available at https://github.com/zhengshijian1993/DC-Net.

## Section 1: Introduction

**Fig 1:**
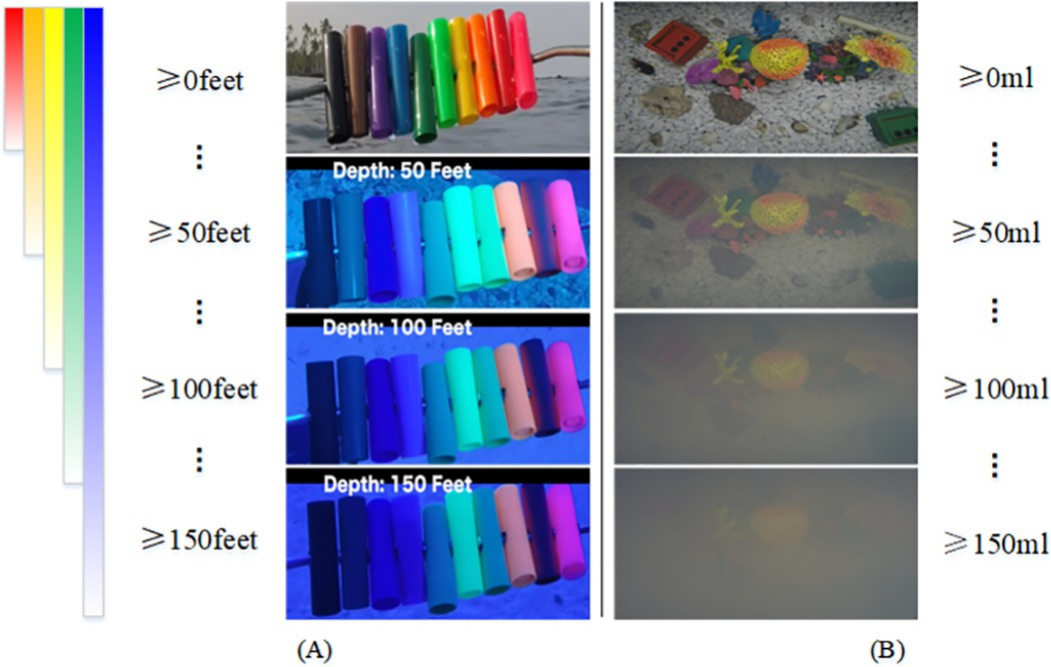
Effects of light absorption and scattering in underwater imaging environments. (A) Light absorption at different water depths, resulting in color deviations. (B) the scattering of light by different turbidity levels of water, resulting in blurred images.

Image enhancement techniques aim to improve the overall or local visual quality of an image, usually as a pre-processing operation for computer vision, which is important for underwater exploration work. Unlike normal outdoor images, underwater imaging environment is quite the special characteristics, and cause image degradation. In-depth studies [1] by some scholars have revealed that this issue is due to the absorption and scattering of light by the water medium, as shown in Fig 1. As a result, underwater images are not captured in a satisfactory result.

**Fig 2:**
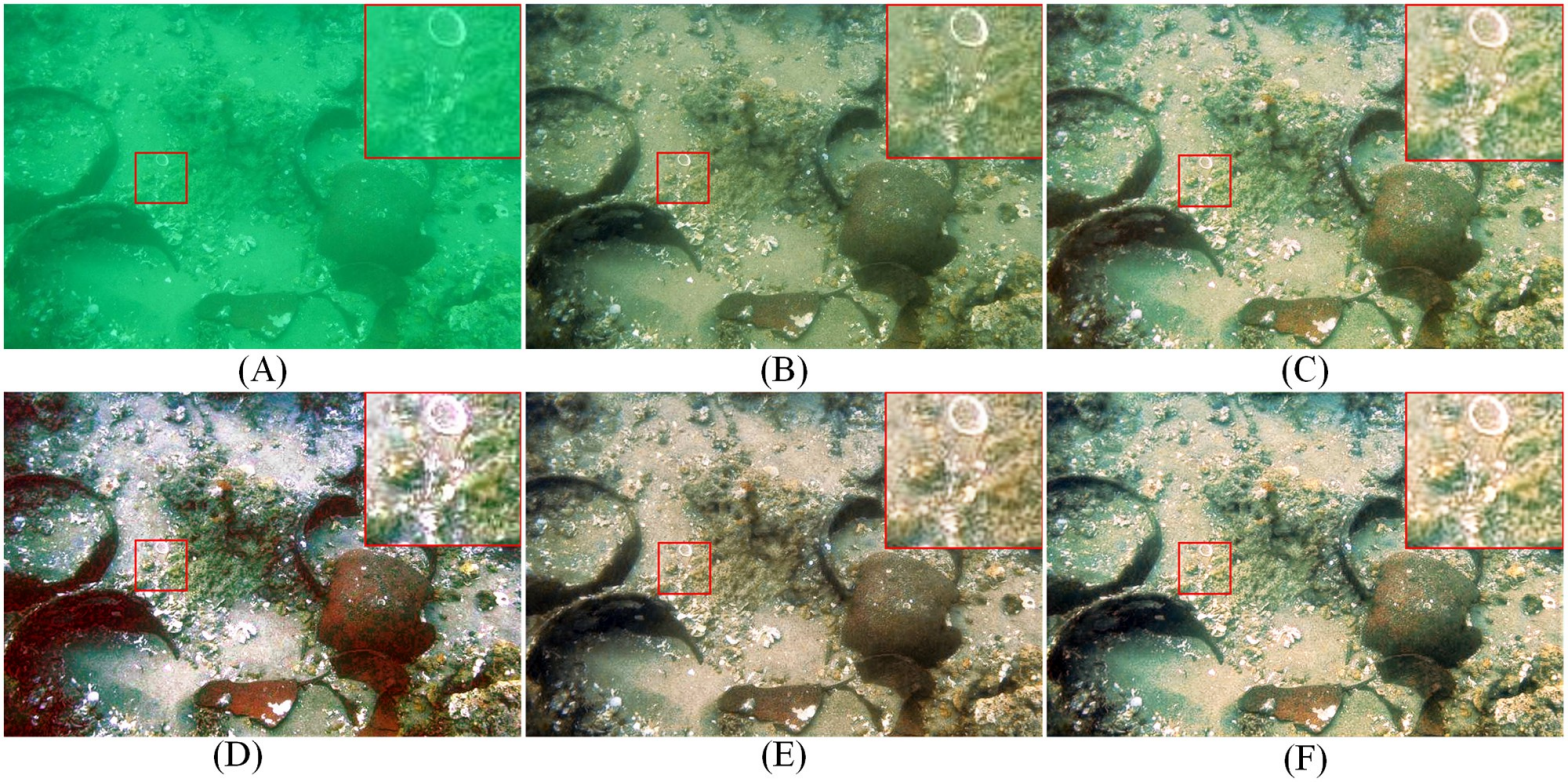
Example of the inherent interference problem between color bias and blurring degradation in underwater images. (A)RAW (B)USLN (C)STSC (D)GT (E)USLN+STSC (F)STSC+USLN.

To solve these problems, a series of underwater image enhancement methods have been proposed to improve the quality of underwater images, most of which go with a single structure to deal with the underwater image degradation problem, and ignore the problem of interference between different degradations. Even though some researchers use dual-stream networks, they do not essentially decompose the task features to deal with them. For example, li et al. [2] designed a medium transmission-guided network for underwater image clarity by considering multiple spatial color information. Wen et al. [3] used physically guided decoupling to predict clear underwater images. These methods use multiple layers of networks to process information, which compensates for the problem of incomplete information between different networks, but do not essentially analyze the problem of interference between multiple degradations of underwater images. An investigation of multiple degradation interference problem with color bias and blurring in underwater images with the statistically guided lightweight underwater image enhancement network (USLN) [4] for partial color feature processing and the Semantic-aware Texture-Structure Feature Collaboration network (STSC) [5] for blur-biased feature processing. The specific results are shown in Fig 2, Fig 2(B) illustrates the color correction process with the single-structure USLN algorithm, showing locally smoother texture features, but the relative boundaries are not well defined. Fig 2(C) presents texture enhancement with the single-structure STSC algorithm, showing locally clear texture features, but the overall color is uneven. Fig 2(E) represents the underwater image successively processed by the USLN and STSC algorithms, showing that there is still an overall color imbalance, but the corresponding image color of the local texture features have been further corrected and also highlights the textural features. Fig 2(F) shows the underwater image successively processed by the STSC and USLN algorithms, showing that there is a weaker local image texture, but the overall color is more balanced. The following problems can be identified 1) Underwater multiple degraded images cannot be handled well using a single-structure approach. 2) Simply working in series for image processing yields results with variability. 3) Texture enhancement algorithms or color enhancement algorithms change the information about the distribution of the image, which affects the further algorithmic processing.

Previous literature [6] has shown that depth feature representations can be used to effectively characterize various image distortions. Ma et al. [7] decomposed image information into multiple frequency bands employing wavelet transforms. Mi et al. [8] decomposed the image into a structural layer with low frequency illumination variance and texture layer features for image enhancement. There is an assumption in this approach that different image degradation information can be decomposed into different frequencies for processing, whereas in practice it is difficult to cleanly separate different degradation factors. Inspired by this theory, we extend it to consider the problem not from a feature-level decomposition of image features, but from different task levels. Based on decomposition theory learning ideas, we design a Divide-and-Conquer network (DC-net) for underwater images, which consists of a texture network based on a multi-axis attention mechanism, a color network based on the look-up tables (LUT) method, and a refinement network. The texture sub-network is an unet structured network based on a multi-axis attention network that captures local and global texture information of an image in both spatial and channel dimensions. The color sub-network uses an adaptive 3D LUT method to extract image color information features and rescale the image range to a color space. The refinement module uses a lightweight convolutional network.

The main contributions of this paper are highlighted as follows:

- We propose a new multi-axis attention module to combine different region/channel features into a single stream structure to extract image features.
- We propose an adaptive 3D lookup tables network to achieve image global color information. In addition, to improve the utilization of the look-up table and memory usage, we reclassify the image range into a more aggregated color space employing a specific gamma curve.
- We have implemented a task decomposition network to solve the problem of hybrid degradation of underwater images. The hybrid degradation of underwater color deviation and blurring is decoupled into two sub-tasks for processing.
- With six no-reference metrics and two reference metrics adopted for the underwater environment, numerous experiments are provided to demonstrate the superiority of the proposed method on both synthesized and real-world underwater images. Finally, we carry out application tests to further show the effectiveness of the DC-net.

## Section 2: Related work

### Underwater image enhancement methods

Underwater image enhancement methods mainly include physical models and data-driven models. Early physical models were mainly built based on atmospheric attenuation models. However, this approach ignored the characteristic that the underwater color channels have different wideband attenuation coefficients. To address this problem, Akkaynak & Treibitz et al. [9] proposed a modified underwater image enhancement model that can obtain better underwater image enhancement results, but considers more prior knowledge and is more computationally complex. To estimate model parameters, some scholars have used deep learning networks to estimate background light and transmission map or scene depth, which relies on the network structure design and training data. In addition, Hao et al. [10] developed an underwater laplace variational model and used luminance mixing and quadratic tree subdivision algorithms to estimate the transmission map and background light. Xie et al. [11] proposed a red channel prior guidance variational framework, which successfully combined the normalized total variational term and the sparse prior knowledge of the fuzzy kernel to achieve better underwater image enhancement results. While this approach can yield satisfactory results, it still does not go beyond the limitations of traditional models (inaccurate parameter estimation). To skip the “pathological problem” of estimating model parameters, many scholars have established underwater image enhancement methods by observing underwater image patterns, such as hyper-laplacian reflectance priors (HLRP) [12], adaptive color and contrast enhancement and denoising (ACCE-D) [13], etc.

In recent years, many studies [14] have shown that deep learning methods work well for low-level vision tasks. Wang et al. [15] used the HSV color space of underwater images to adjust the underwater image brightness, color, and saturation and the RGB color space to denoise and remove color bias to obtain high-quality underwater images. The method analysed the input space containing different feature information and the enhanced image faithfully represents the original underwater image. Qi et al. [16] proposed a new underwater image enhancement architecture by using the semantic information introduced by region feature learning as a high-level guide. The approach brought semantic consistency and visual image enhancement from network architecture analysis. Gao et al. [17] used multi-scale dense generative adversarial networks to enhance underwater images, and the enhanced images improve the perceptual quality of the images. Physical models and deep learning methods each have advantages and disadvantages for underwater image enhancement processing, Zhou et al. [18] proposed a new framework by integrating the physical model, domain adaptation, and generative adversarial networks(GAN) with feedback control ideas. The authors made the physical model constrain the estimation of the GAN framework and used the physical model as the feedback controller of the GAN-enhanced network to provide definite constraints for ill-posed problems and ensured that the estimation results are consistent with the observed images.

### Decomposition feature learning

Decoupled representation learning is a way to be able to decompose the varying factors in the feature representation into mutually independent parts for processing under certain conditions. In previous studies, decoupled representation learning has been widely used in various computer vision task weights in areas such as domain adaptation, zero-sample learning, and scene graph generation [19]. These are mostly low-level perception tasks in which decoupled learning plays the role of guidance and foundation. A common form is that decoupled information is used as a basis for subsequent tasks, which improves the accuracy of the algorithm. Bianco et al. [6] analyzed deep visual representations to essentially characterize different types of image distortions and showed that a given number of network layers allow for efficient separation of different types of distorted feature spaces. Based on this research theory, many scholars have introduced the decoupling approach to the image degradation problem. Such as liu et al. [20] decomposed the image into different frequency domains for removing the moiré problem via wavelet transform. Although the authors effectively got better results by wavelet frequency domain decomposition processing, the wavelet decomposition was designed by specific formulae, which is at variance with the related task. To this end, Wang et al. [21] designed three types of spatial, angular, and polar plane decoupling convolutions to decouple the optical field into a two-dimensional subspace, and then solved the super-resolution and parallax estimation problems by designing the relevant modules to fuse the information extracted from different subspaces. However, this feature decoupling by convolution makes it difficult to quantify the effectiveness of decoupling due to the uncertainty of the lost features of the convolution. li et al. [22] used gain control-based normalization to achieve separation of different distortion features, adaptive filtering of distortion representations, and aggregation of useful content information to obtain image enhancement results. However, the above methods that provide guidance and foundations for deep models, i.e., the features used for learning are all just feature layer decompositions and not task-oriented features. To provide more specific information, there is an urgent need for a decoupled task-oriented feature learning strategy.

## Section 3: Materials and methods

To address the issues mentioned in section 1, we proposed a Divide-and-Conquer network framework for underwater image enhancement, i.e, DC-net. Specifically, we designed a branch of the underwater texture enhancement network with a multi-axial attention mechanism and a branch of the underwater color correction network with a LUT mechanism. In the following subsections, the overall structure and key modules will be described in detail.

### Ethical statement

The datasets used in our experiment are publicly available datasets. The experiment did not involve critically ill animals or protected species, so no relevant permits are required for the experiment.

### Overall network with decomposition feature learning

**Fig 3:**
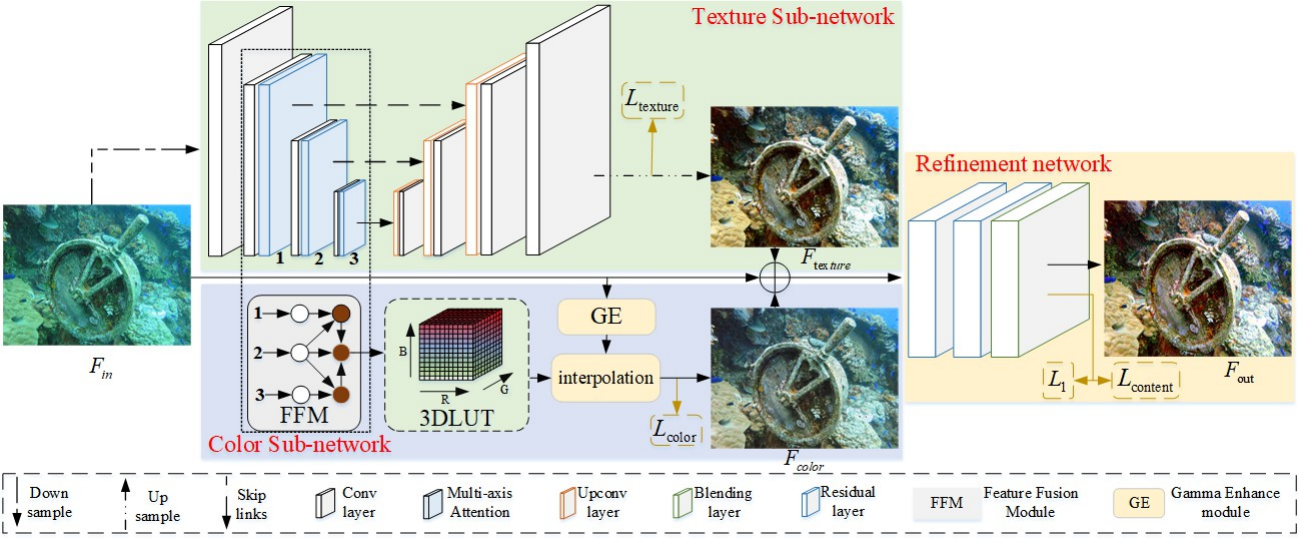
The illustration of our network for image enhancement. Our network contains he texture sub-network, the color sub-network, and the refinement network.

The overall framework of our proposed the DC-net method is presented in Fig 3, which mainly consists of the texture sub-network, the color sub-network and the refinement network. In the texture sub-network, the input image (*F*_*in*_) is first fed to the encoder that introduces the multi-axis attention module to extract texture features, which is then decoded to obtain a texture-enhanced image(*F*_*texture*_), and the final result is fed to the fusion network. In the color sub-network, the multi-level image features obtained from the texture network encoder are first fused and processed as input to the 3D LUT module. At the same time, the underwater image is fed into the image-specific gamma curve module to obtain more focused image color information. We then interpolate the image color information features on the LUT to obtain a color-enhanced image(*F*_*color*_), and the result is fed to the fusion network. Finally, the results of the texture network and the color network are fused with the input image to obtain the underwater image enhancement results (*F*_*out*_). The DC-net method can be described as follows

$$\begin{array}{c} F_{texture}=Tex\left(F_{in}\right) \end{array}$$ (1)

$$\begin{array}{c} F_{color}=Col\left(F_{in}\right) \end{array}$$ (2)

$$\begin{array}{c} F_{out}=Fusion\left(F_{in},F_{texture},F_{color}\right) \end{array}$$ (3)

where *Fusion* denotes the refinement network. *Tex* denotes the texture sub-network. *Col* denotes the color sub-network.

### Texture sub-network

**Fig 4:**
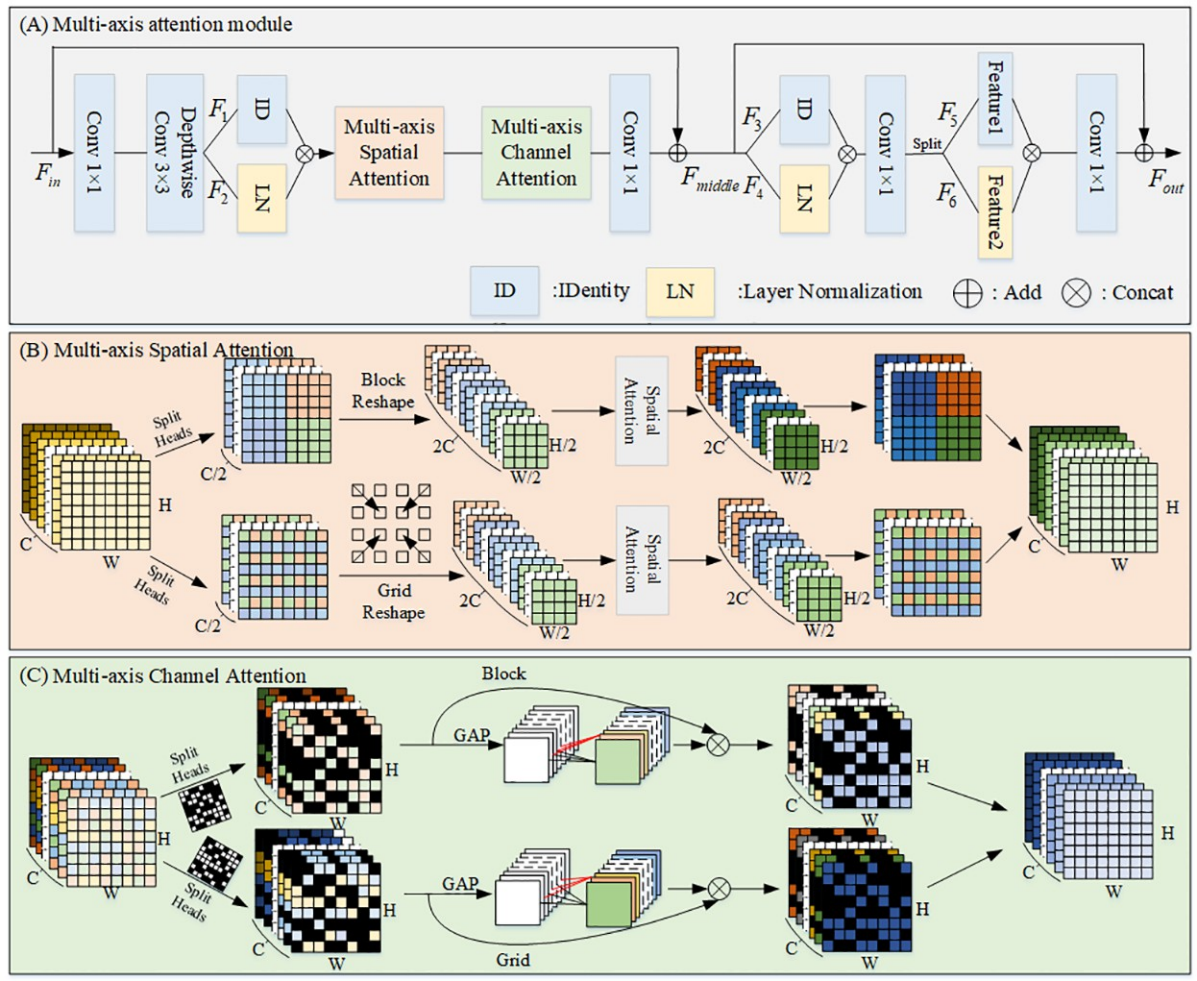
Multi-axis attention module.

The texture sub-network is constructed based on the unet network model, however, ordinary convolution is not able to extract image texture information effectively, so the multi-axis attention module is proposed to increase the feature representation and channel variant capability of ordinary convolution. Our work is inspired by the multi-axis block proposed in Axial attention [23, 24], which performs attention on multiple axes, and we find that the block is the key to achieving significant performance improvement in the experiment. However, the method only considers the channel axis decomposition and not the spatial axis decomposition. Therefore, we design a channel attention mechanism for spatial multi-axis processing based on this approach, As shown in Fig 4(A). Multi-axis attention module first takes the input feature $F_{in}\in R^{H\times W\times C}$ and applies 1 × 1 convolutions and 3 × 3 depth-wise convolutions to encode features. Then, the features are divided into two parts, one of which is normalized by Layer Normalization (LN) of the parameters, and connected to the other part of the features. Afterward, the features are reinforced by Multi-axis Channel Attention (MCA) and Multi-axis Spatial Attention (MSA), and the channel information is adjusted by 1 × 1 convolution. The features ($F_{middle}\in R^{H\times W\times C}$) by adding the feature with shortcut features (*F*_*in*_). To transform features, the features are divided into two parts, one of which is normalized by Layer Normalization of the parameters, and connected to the other part of the features. The channel information is adjusted by 1 × 1 convolution. To increase the non-linearity of the image feature extraction, we decompose the features into two parts and multiply the two parts to obtain the hybrid image features. The channel information is adjusted by 1 × 1 convolution. The features ($F_{out}\in R^{H\times W\times C}$) by adding the feature with shortcut features (*F*_*middle*_). The multi-axis attention module can be described as follows

$$\begin{array}{c} F_{1},F_{2}=split\left(C_{3}\left(C_{1}\left(F_{in}\right)\right)\right) \end{array}$$ (4)

$$\begin{array}{c} F_{middle}=C_{1}\left(MCA\left(MSA\left(connect\left(LN\left(F_{1}\right),ID\left(F_{2}\right)\right)\right)\right)\right)+F_{in} \end{array}$$ (5)

$$\begin{array}{c} F_{3},F_{4}=split\left(F_{middle}\right) \end{array}$$ (6)

$$\begin{array}{c} F_{5},F_{6}=split\left(C_{1}\left(connect\left(LN\left(F_{3}\right),ID\left(F_{4}\right)\right)\right)\right) \end{array}$$ (7)

$$\begin{array}{c} F_{out}=F_{middle}+C_{1}\left(connect\left(F_{5},F_{6}\right)\right) \end{array}$$ (8)

where *C*_1_ and *C*_3_ denote 1 × 1 convolution and 3 × 3 depth-wise convolutions. *LN* denotes Layer Normalization. *ID* denotes identity connection. Connect denotes splicing of two features. Split denotes decomposition of the features along a channel into two equal channels of features. *MCA* denotes Multi-axis Channel Attention, and *MSA* denotes Multi-axis Spatial Attention.

#### Multi-axis Spatial Attention

As shown in Fig 4(B). The input feature map is first split into two separate heads along the image feature channel, processed by the global and local paths respectively. The local path is shown in the upper branch of Fig 4(B), where use a fixed window grid partitions the feature map and feeds it into the spatial attention model to enhance the image spatial feature extraction information, and then obtain the local feature extraction information in the reverse aggregation results. The global path is shown in the lower branch of Fig 4(B), the same operation as in the local branch is used to obtain global feature information for the image, except that a dilated grid is used instead of a fixed window grid. Finally, we aggregate the image’s local features and global features to obtain the image enhancement results.

#### Multi-axis Channel Attention

As shown in Fig 4(C). The input feature map is first split into two separate heads along the image space feature (H-axis), which are processed by the global and local paths respectively. The main operation here is to divide the input feature image into small feature maps and randomly mask half of the small feature map blocks along the H-axis to stitch together, thus obtaining two *H*/2 feature maps split along the H-axis. The local path is shown in the upper branch of Fig 4(C). We first pool the segmented feature maps globally on average, then calculate the fixed channel blocks by fast 1D convolution with a kernel size of 4 to obtain the enhanced channel weights after the enhancement, and multiply this weight with the segmented feature maps to obtain the channel-enhanced feature maps. The global path is shown in the lower branch of Fig 4(C), the same operation as the local branch, except that the fixed channel blocks are replaced using the interval channel blocks to obtain the global feature image of the global image. Finally, we aggregate the local and global features of the image to obtain the image enhancement result.

### Color sub-network

**Fig 5:**
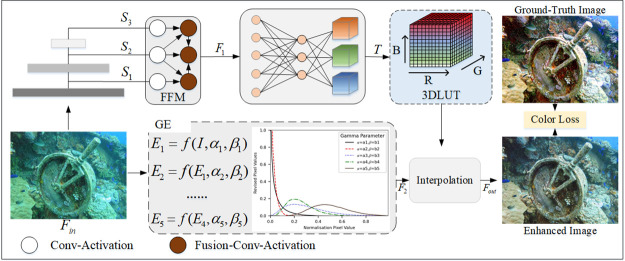
Framework of color transformation network.

To correct color deviations in underwater image, we propose a color sub-network based on the LUTs color feature extraction module, as shown in Fig 5. The traditional LUTs is a set of image editing tools for professional color shifting through a two-step process of look-up and interpolation, which can influence parameters such as hue, saturation, and luminance in a fully stereoscopic color space control to change the color. However, this method requires manual design and fixed parameters and is not very scalable. To solve the problem, we use a set of learnable LUTs as the base transform to cover the color transformation space, with the learnable LUTs being learned automatically by a neural network. To exploit the multi-scale information, we propose a feature fusion model (FFM) to enhance the image features, as shown in the FFM module in Fig 5. In addition, In response to the low utilization of LUTs units. Influenced by recent developments in neurology [30], we propose the gamma enhance (GE) that iteratively approximates pixel-level and higher-order curves that can redistribute and normalize the input image range into color space, improving unit utilization, as shown in the GE module in Fig 5. The color sub-network first takes the input feature $F_{in}\in R^{H\times W\times C}$ and applies multi-layer convolution to encode the image features to obtain *S*_1_, *S*_2_, *S*_3_ features. These features are processed in two branches, the first branch passes the features through the fused features module to obtain the *F*_1_ features and through the fully connected layer to obtain the 3D RGB color values (*T*). The second branch takes the features through the gamma enhancement module to obtain more aggregated *F*_2_ features in color space. Finally, the *F*_2_ features are interpolated in the 3D color table of T to obtain the color-enhanced image (*F*_*out*_). The color sub-network can be described as follows

$$\begin{array}{c} S_{1},S_{2},S_{3}=E\left(F_{in}\right) \end{array}$$ (9)

$$\begin{array}{c} T=FCL(FFM\left(S_{1},S_{2},S_{3}\right)) \end{array}$$ (10)

$$\begin{array}{c} F_{2}=GE\left(S_{1},S_{2},S_{3}\right) \end{array}$$ (11)

$$\begin{array}{c} F_{out}=Interpolation\left(F_{2},T\right) \end{array}$$ (12)

where *E* denotes a feature extraction network, *FFM* denotes a feature fusion module, *FCL* denotes a fully connected layer, *GE* denotes a gamma curve enhancement module, and *Interpolation* denotes a trilinear-interpolation approach.

#### Feature fusion module

As shown in the FFM in Fig 5, a node with a single input represents a layer of convolution and activation function, and a node with multiple inputs represents a fusion layer, convolution layer and activation function. The fusion layer can be described as follows

$$\begin{array}{c} F_{O}=\sum_{i=1}^{M}\frac{w_{i}}{\sum_{j}w_{j}}\cdot I_{i} \end{array}$$ (13)

Where *w*_*i*_ is a weight for the *i* − *th* input *I*_*i*_. *M* denotes the number of inputs. *F*_*O*_ indicates fused features.

#### Gamma enhance module

Inspired by curves to adjust image information [25], we have tried to design a curve that can automatically map an underwater image to its enhanced version. It can be expressed as

$$\begin{array}{c} E(I(x),\alpha ,\beta )=I(x)+\alpha I(x)(1-I(x))+\beta I(x)(1-I(x)) \end{array}$$ (14)

where *α* and *β* denote trainable curve parameters, *I*(*x*) denotes a given input image. *E*(*I*(*x*), *α*, *β*) denotes the enhanced image features.

Although this curve enables the image to be adjusted over a wider dynamic range, it is still a global adjustment (*α* and *β* for all pixels). We formulate *α* and *β* as per-pixel parameters, i.e. each pixel of a given input image has a corresponding enhancement curve. Here the formulae are represented by the *C*_1_ and *C*_3_ feature maps. The higher-order iterative curve formula can be expressed as

$$\begin{array}{c} E_{n}=E_{(}n-1)\left(x\right)+A_{n}\left(x\right)E_{(}n-1)\left(x\right)\left(1-E_{(}n-1\right)\left(x\right))+B_{n}\left(x\right)E_{(}n-1)\left(x\right)\left(1-E_{(}n-1\right)\left(x\right)) \end{array}$$ (15)

where *A*_*n*_(*x*) and *B*_*n*_(*x*) are a parametric map of the same size as the input image. *E*_*n*_ denotes the *n* − *th* enhanced image.

### Refinement network

The texture sub-network and color sub-network are transformed into richly detailed features. However, simply mixing texture network features with color features would not achieve the desired result, so we design a lightweight refinement module to produce better results.

Our refinement module consists of two convolutional layers and an image blending layer. Specifically, we first up-sample the texture sub-network enhanced image and the color sub-network enhancement image to the same size, and then input the original input image, the texture enhancement result and the color enhancement result into the convolution module and the image blending layer to obtain the underwater enhanced image.

#### Image blending layer

The image blending layer is described as follows, we firstly connect the input image (*F*_*in*_), texture enhancement result image (*F*_*texture*_) and color enhancement result image (*F*_*color*_) and obtain the image feature weights (*W*). Then the weights are multiplied with the texture enhancement result image and color enhancement result image respectively. Finally, we add the results to get the blended image (*F*_*out*_). The image blending layer can be described as follows

$$\begin{array}{c} F_{f}=connect\left(F_{in},F_{texture},F_{color}\right) \end{array}$$ (16)

$$\begin{array}{c} W=sigmoid(C_{1}\left(relu\left(C_{3}\left(F_{f}\right)\right)\right)) \end{array}$$ (17)

$$\begin{array}{c} F_{out}=W*F_{texture}+W*F_{color} \end{array}$$ (18)

where *C*_1_ and *C*_3_ denote 1 × 1 convolution and 3 × 3 convolutions.

### Loss function

The proposed DC-net network uses a pairwise image supervised training strategy, where the combined loss includes four sub-loss functions, L1 loss, Content loss, Color loss and Texture loss. The *J*_*F*_ indicates the predicted image, the *J*_*T*_ denotes the ground-truth image. the L1 loss presented in equation:

$$\begin{array}{c} l_{l1}={\left\|J_{F}-J_{T}\right\|}_{1} \end{array}$$ (19)

The Perceptual loss [26] is a comparison of the eigenvalues obtained by convolving the *J*_*F*_ with the *J*_*T*_, making the image more similar in terms of high-level information. The perceptual loss function is as follows:

$$\begin{array}{c} l_{\text{content}\;}=\frac{1}{c_{j}H_{j}W_{j}}\|\varphi_{j}(F_{w}(J_{F}))-\varphi_{j}(J_{T})\| \end{array}$$ (20)

where *c*_*j*_,*H*_*j*_ and *W*_*j*_ represents the number, height and width of the feature maps, *F*_*W*_(*J*_*F*_) represents the enhanced image.

The color loss function compares the *J*_*F*_ with the *J*_*T*_ in terms of angular and distance features. Specifically, the color information of the *J*_*F*_ and the *J*_*T*_ is first obtained by Gaussian blurring [27], then the distance [28] and angle [29] differences between the two images are calculated, and finally the two are combined linearly. The colour loss function formula is as follows:

$$\begin{array}{c} l_{p}=\|{\left(\Delta R-\Delta G\right)}^{2}+{\left(\frac{\Delta R+\Delta G}{2}-\Delta B\right)}^{2}{\|}_{2} \end{array}$$ (21)

$$\begin{array}{c} l_{a}=\sum_{p}\nabla ((F(J_{F}){)}_{p},(J_{T}{)}_{p}) \end{array}$$ (22)

$$\begin{array}{c} l_{color}=0.25l_{p}+0.75l_{a} \end{array}$$ (23)

Where Δ*R*,Δ*G* and Δ*B* represent the difference between the red, green and blue channels of the predicted image and the ground-truth image. ∇() indicates the angle operator between two image colors.

The texture loss function is a comparison of predicted grey-scale images and real grey-scale images by CNN feature values, mainly inspired by the literature, where image texture information is related to the spatial distribution of grey levels [30]. We define the texture loss function as:

$$\begin{array}{c} l_{\text{texture}}=\|G(\varphi (J_{F}))-G(\varphi (J_{T})){\|}_{2}^{2} \end{array}$$ (24)

The final combination loss is a linear combination of L1 loss, perceptual loss, Color loss and Texture loss:

$$\begin{array}{c} L=\alpha_{1}l_{l_{1}}+\alpha_{2}l_{\text{content}}+\alpha_{3}l_{\text{color}}+\alpha_{4}l_{\text{texture}} \end{array}$$ (25)

Where *α*_1_, *α*_2_, *α*_3_ and *α*_4_ are generally set empirically to balance different losses. We experimentally set *α*_1_ = 1,*α*_2_ = 10, *α*_3_ = 0.5 and *α*_4_ = 0.5.

## Section 4: Experimental results

### Baseline methods

To demonstrate the effectiveness of our proposed method, extensive experiments were conducted on different datasets between DC-net and 10 SOTA underwater image enhancement algorithms. In more detail, four representative traditional methods were selected for comparison, including transmission estimation in underwater single images (**UDCP**) [31], initial results in underwater single image dehazing (**MIP**) [32], underwater image enhancement with a deep residual framework (**CLAHE**) [33], color balance and fusion (**CBF**) [34]. Our method was also compared with six deep learning-based methods, wavelet-based dual-stream network (**UIE-WD**) [7], contrastive underwater restoration (**CWR**) [35], representative color transform network (**RCT**) [36], a statistically guided lightweight underwater image enhancement network (**USLN**) [4], semantic-aware texture-structure feature collaboration (**STSC**) [5], contrastive semi-supervised learning for underwater image restoration (**Semi-UIR**) [37].

### Implementation details

Our model is trained for a total of 200 epochs. All deep learning models are optimized using the Adam optimizer. The initial learning rate is 1e-2, which will be halved at every 50 iterations. The parameters *β*_1_ and *β*_2_ in the optimizer take the default values, i.e., 0.9 and 0.999. We performed data augmentation by mixup. The batch size is set to 4. All test images are fed into the model with their full resolution to generate enhanced images during the evaluation procedure. The configurations of the training server are described as 1 Intel Core i7–6800 K processors, 1 NVIDIA Titan RTX GPUs (24 GB), 64-GB memory, and an Ubuntu 16.04 operating system.

### Dataset and evaluation metrics

Our method was tested on the underwater image enhancement benchmark (**UIEB**) [38], synthetic underwater image dataset (**SUID**) [39], synthetic underwater image dataset 1 (**SUID1**) [40], and underwater image enhancement for improved visual perception dataset (**EUVP**) [41]. The UIEB datasets contains a datasets of 950 real-world underwater images that contain a distinct variety of underwater image quality degradation features (e.g. color bias, low contrast, blurred detail, etc.). The ground truth images are created by selecting the results of 12 underwater image enhancement methods by 50 volunteers. The SUID datasets contains 4000 synthetic underwater images. The datasets is based on the NYU-v2 datasets and generates synthetic underwater datasets based on different attenuation absorption rates for different types of water (10 types). Each of these water types are synthesized into 200 images. The SUID1 datasets is developed based on an underwater imaging model and underwater optical transmission characteristics, and a total of 900 underwater images are synthesized, including four types of scenes: green light, blue light, low light and blur. The EUVP paired datasets consists of three subsets of subjects, here we focus on the image-net subset, which contains 8670 underwater images, and the datasets is mainly composed using images captured by seven different cameras at different visibility and locations, as well as images intercept from online videos. The ground truth images are composed of the optimal results of multiple underwater enhancement methods selected by the observer.

To train the DC-net network, we randomly divided the UIEB datasets, EUVP datasets, SUID datasets and SUID1 datasets into 1:2:7 validation, testing and training subsets. We chose the UIEB datasets and SUID datasets to construct the training set of 3465 images. For testing, we validated each of the four subsets of the datasets set of sliced test sets.

We used eight image quality evaluation metrics, namely Peak Signal Noise Ratio (**PSNR**), Structural Similarity (**SSIM**), underwater image quality metric (**UIQM**) [42], underwater color image quality evaluation (**UCIQE**) [43], Twice Mixing (**TM**) [44], A combination index of Colorfulness, Contrast and Fog density (**CCF**) [45], **Entropy**, Natural Image Quality Evaluator (**NIQE**) [46]. The higher the PSNR and SSIM score, the better the enhanced image. UIQM includes three attribute measures: colorfulness, sharpness, and contrast measures. The UCIQE uses a linear combination of chromaticity, saturation, and contrast for quantitative assessment, quantifying uneven color bias, blur, and low contrast respectively. The TM evaluates image quality by using two blending ratios in the generation of training data and the supervised training process. The CCF is a feature-weighted metric with a combing colorfulness index, contrast index, and fog density index, which can quantify color loss, blurring, and fog, respectively. The entropy indicates the entropy value of the image. NIQE is based on a set of ‘quality-aware’ features, which are fitted to an MVG model.

### Comprehensive comparisons on the real-world underwater images dataset

**Fig 6:**
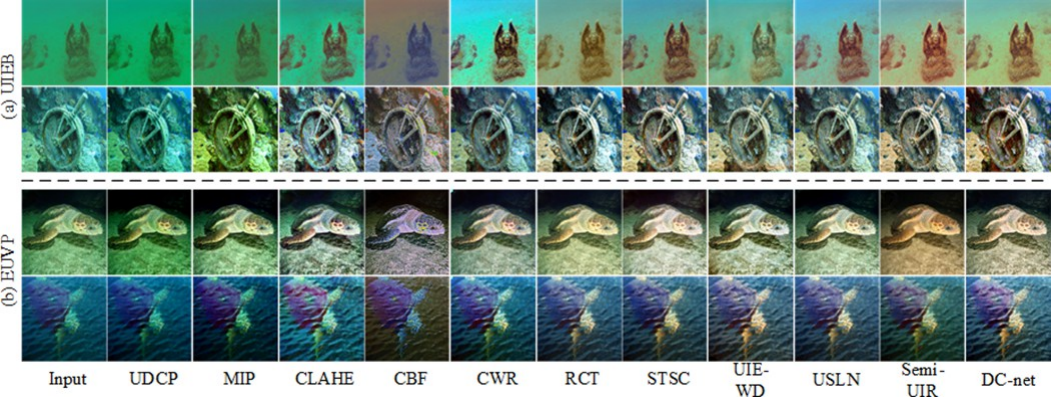
Visualization of the comparision results for the UIEB and EUVP dataset.

1) Qualitative comparisons: We validate the method on two real environment underwater image datasets (UIEB and EUVP datasets), as shown in Fig 6, the results obtained from the Physical model-based methods are unsatisfactory, such the UDCP method causes the image darker, the MIP and CLAHE method produce the color unevenness of the image, and the CBF method produce over-processed images. The images obtain from data-driven methods can get better results, yet through careful comparison, we find that there is still a certain gap between these methods. The CWR method provides a limited improvement in contrast and color balance in underwater images. The RCT method can obtain high contrast and sharp images, but cannot handle underwater bluish images. The STSC method can enhance detailed texture information, but there is an image color imbalance. The UIE-WD method is not effective in correcting colour deviation areas. The USLN method is effective in correcting underwater image colors, but is somewhat over-processed (the processed image is bluish). The Semi-UIR method is essentially the experimental deep learning method that shows the best results, however, the color treatment is still a little on the dark side and some of the image texture detail is excessively smooth. Compared to the SOTA method, our proposed DC-net method obtains optimal results.

2) Quantitative comparisons: To further illustrate the superiority of the proposed DC-net network, we quantitatively compared the DC-net network with several SOTA methods, and Table 1 shows the average scores of the test set of enhanced images. We observed that (1) the physical model approach (UDCP, MIP) received lower scores, but not necessarily the physical model approach (CLAHE) scored worse than the deep learning approach. (2) The most SOTA methods can only achieve optimal or sub-optimal scores on one indicator. (3) Our proposed DC-net method is capable of achieving optimal or sub-optimal scores on multiple indicators.

**Table 1:** Underwater image quality evaluation of different enhancement methods on real world underwater image. The best results are marked in bold.

Dataset	Method	UDCP	MIP	CLAHE	CBF	CWR	STSC	RCT	USLN	UIE-WD	Semi-UIR	DC-net
UIEB	PSNR	16.7919	17.8031	20.4559	17.5358	24.2923	24.4637	21.1268	20.6428	25.5452	**26.6932**	25.7896
	SSIM	0.7013	0.7061	0.8424	0.5593	0.8599	0.8767	0.7327	0.8662	0.8948	0.9162	**0.9234**
	UCIQE	5.1428	5.8425	4.4639	4.9489	4.8876	4.9528	4.6332	4.6029	4.6444	4.7619	**5.8652**
	UIQM	0.3597	0.8318	0.8893	0.9900	0.7868	0.7752	0.8820	0.8448	0.7696	0.9061	**1.1365**
	NIQE	4.3192	3.4302	3.3866	7.3073	4.8359	3.5843	3.4521	3.4164	4.1452	4.7917	**3.0237**
	CCF	15.1024	16.8057	24.2353	25.9499	20.7870	29.2773	27.0381	24.7450	29.8479	**33.8201**	29.9591
	Entropy	6.6726	6.7001	7.5984	7.3671	7.6141	7.6651	7.6716	7.5914	7.6076	7.7285	**7.7608**
	TM	0.6095	0.9869	1.3232	0.2516	1.0789	1.3111	1.2415	1.3457	0.9172	2.6749	**2.8847**
EUVP	PSNR	21.0929	18.5540	19.3063	19.6040	22.3492	21.4512	20.9443	21.1535	22.3269	21.3406	**22.8644**
	SSIM	0.63097	0.5969	0.6739	0.5168	0.7355	0.7429	0.7519	0.7433	0.7672	0.7228	**0.7686**
	UCIQE	5.3084	6.6723	4.9955	**5.6333**	4.6934	4.3411	4.2699	4.1550	4.1247	3.7065	4.0983
	UIQM	0.2749	0.6521	0.7413	**1.3818**	0.5099	0.6618	0.7342	0.6566	0.6266	0.8234	0.8799
	NIQE	4.8112	5.1773	4.9955	7.8387	4.9757	4.8586	4.9828	**4.5724**	5.0132	5.7554	4.7856
	CCF	18.1335	19.6725	24.1538	23.2991	25.7165	32.6928	30.7340	30.6621	30.8694	**34.6832**	30.5613
	Entropy	7.1441	6.9491	7.7634	7.4891	7.6125	7.6754	7.6661	7.6729	7.6456	7.7194	**7.8472**
	TM	0.9428	1.0104	1.2058	0.3008	1.1972	1.4833	1.4311	1.5141	1.4088	1.4239	**1.5374**

### Comprehensive comparisons on the synthetic underwater dataset

**Fig 7:**
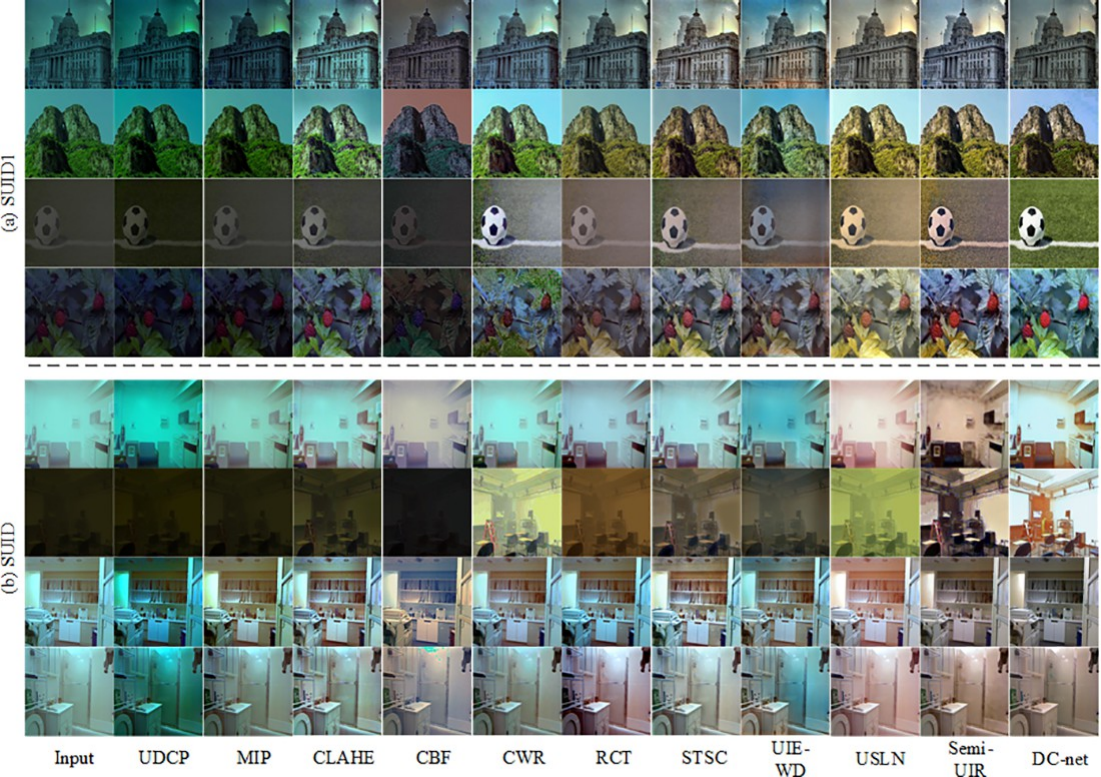
Visualization of the comparision results for the SUID and SUID1 dataset.

1) Qualitative comparisons: We validated the superiority of our method on two underwater image datasets (SUID and SUID1 datasets) in a synthetic environment. As shown in Fig 7, the first to fourth rows show the results for the SUID1 datasets, specifically, the first row shows the results for the bluish datasets, the second row shows the results for the greenish datasets, the third row shows the results for the hazy datasets and the fourth row shows the results for the low-light datasets. The fifth to eighth rows show the results of the SUID datasets, specifically, the fifth row shows the type1 data result, the sixth row shows the type7 data result, the seventh row shows the type-IA data result and the eighth row shows the type-II data result. Overall, the results handled by the deep learning methods are better than those of the conventional methods. The UDCP method is largely unable to deal with underwater images on the SUID1 datasets and increases the color deviation of different types of data on the SUID datasets. The MIP method is largely unable to treat underwater images in the SUID1 datasets and is able to handle underwater images with small degradation but did not have large degradation in the SUID datasets. The CLAHE method was able to obtain corrected results on both datasets but did not handle the results of the large degradation underwater images very well. The CBF method is able to correct the small degraded underwater images, but at the same time makes the results of the large degraded underwater images worse, in addition to the large color deviations recovered. The CWR method can deal with all kinds of underwater degradation, but there are some deviations in the results for underwater images with large degradation obtained by the different methods, indicating that the different ways of composing underwater images affect the results of the subsequent algorithms. The RCT method can handle the degradation of underwater images but with some color distortion. The STSC method is capable of showing the details of the underwater images, but the color recovery is imbalanced. The UIE-WD method is better on the SUID1 datasets, but the images are bluish on the SUID datasets. The USLN method is somewhat over-processed, with some areas of the small degraded images in the SUID datasets excessively enhanced and the large degraded underwater images greenish in color. There is a layer of haze in the low-light treated images in the SUID1 datasets. The Semi-UIR method gives the best results for the deep learning method, but the local texture is relatively unsmooth and the color recovery is unbalanced in some areas. Compared to the SOTA method, our proposed DC-net method obtains optimal results.

2) Quantitative comparisons: To demonstrate the superiority of the propose DC-net network for multiple types of underwater images, we quantitatively compare the SOTA method. Table 2 shows the average scores for the enhance image test set, with bold markers indicating optimal results. We observe that (1) Underwater image enhancement methods have different enhancement results for datasets synthetic by different methods (SUID and SUID1). Multiple SOTA methods scored lower on test sets of multiple types of blended underwater images. (2) The propose DC-net method does not necessarily work better on the synthetic dataset than on the real dataset, possibly due to multiple types of water images (different image domains). (3) Our propose DC-net method scores highest than the current SOTA method on several metrics.

**Table 2:** Underwater image quality evaluation of different enhancement methods on synthetic underwater image. The best results are marked in bold.

Dataset	Method	UDCP	MIP	CLAHE	CBF	CWR	STSC	RCT	USLN	UIE-WD	Semi-UIR	DC-net
SUID	PSNR	14.5019	13.9203	14.8259	14.4121	16.2471	18.7013	13.3979	19.5514	13.9377	21.5274	**23.5689**
	SSIM	0.5447	0.6047	0.6600	0.5622	0.7017	0.7599	0.4078	0.8048	0.7076	0.7718	**0.8924**
	UCIQE	4.9091	4.2759	3.5572	3.2478	4.1631	2.9898	3.5046	3.8039	3.8761	3.7686	**5.8142**
	UIQM	0.1034	0.3273	0.5329	0.6181	0.6177	0.4247	0.6594	0.4948	0.7582	0.6178	**1.0562**
	NIQE	4.8422	5.0255	4.3473	7.3315	4.9020	4.6070	3.4997	4.1691	4.1190	5.0392	**3.0257**
	CCF	23.9036	27.7786	18.1895	24.0018	17.1392	21.7244	21.9391	17.8568	18.2202	**32.9985**	27.7878
	Entropy	6.5192	6.3131	6.9827	6.4717	7.3101	6.9391	7.1755	7.1478	7.0309	**7.4899**	6.9424
	TM	0.3581	0.4068	0.4925	0.2545	0.5246	0.5217	0.4946	0.4926	0.5327	**0.9872**	0.6671
SUID1	PSNR	9.7133	10.2424	15.6405	10.4744	**23.1442**	19.0151	15.2545	17.4636	18.1117	21.2159	17.4696
	SSIM	0.4398	0.4906	0.8045	0.5591	0.8575	0.8370	0.4033	0.8029	0.8831	0.8248	**0.8927**
	UCIQE	4.2731	3.4564	4.0738	2.0667	4.4481	2.7441	3.4401	3.1109	**5.6343**	4.0839	4.9095
	UIQM	0.2155	0.1834	0.4902	0.8349	0.7723	0.6297	0.8958	0.6706	1.1650	**1.2052**	0.7316
	NIQE	4.0451	4.1703	4.0014	9.8531	3.8408	4.0439	3.6395	3.6606	3.8414	5.0353	**3.4743**
	CCF	22.3801	27.7787	21.8966	25.4535	23.3458	20.0900	23.8327	20.8888	24.2148	**31.1499**	29.9331
	Entropy	6.3285	6.5752	7.0813	6.4653	7.4254	6.9102	7.1917	7.1478	7.3515	7.4236	**7.6184**
	TM	0.7146	0.6936	1.1323	0.2312	1.3201	1.1840	1.2720	1.2571	1.3820	**2.1674**	1.2258

### Ablation study

We designed ablation experiments on the UIEB and SUID1 dataset. All experiments were trained for 200 epochs by default, with the same model parameter settings.

#### Effectiveness of the texture sub-network

**Fig 8:**
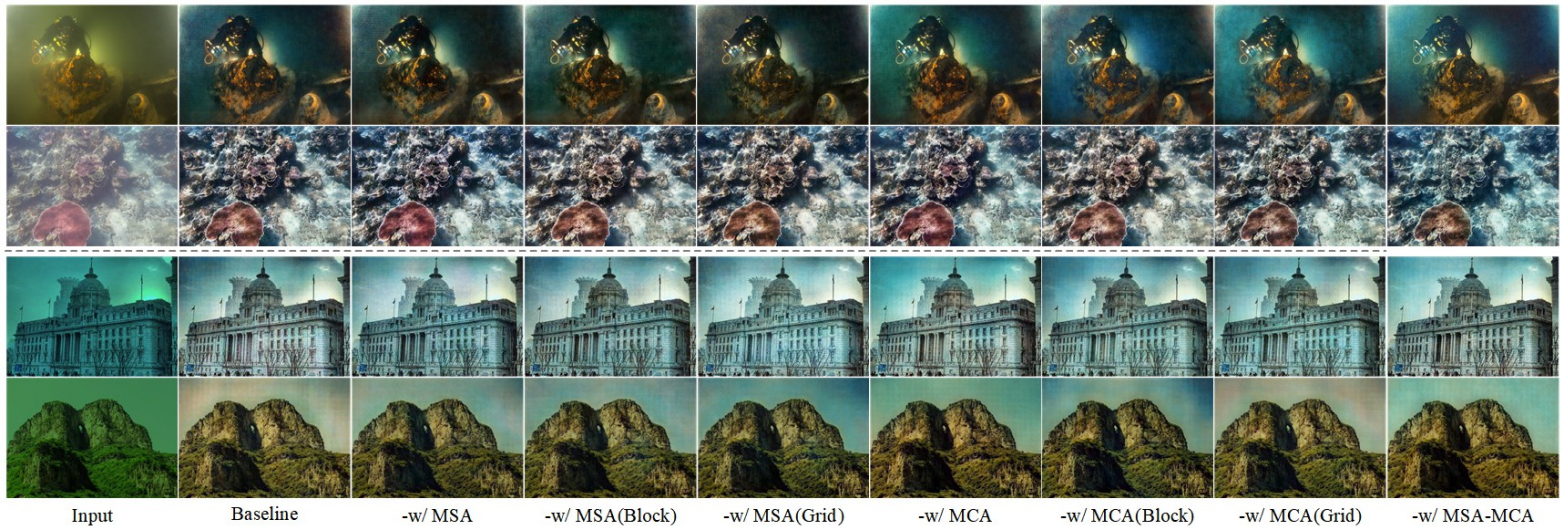
Qualitative ablation results for each key component of the texture sub-network.

First of all, we use the network of the unet-like as a baseline network and train it with the reference loss of the texture. We add different components to the baseline network, i.e. (a) -w/ MSA, which adds MSA to the baseline network. (b) -w/ MSA(Block), which adds the block branch of the MSA to the baseline network. (c) -w/ MSA(Grid), which adds the grid branch of the MSA to the baseline network. (d) -w/ MCA, which adds the MCA to the baseline network. (e) -w/ MCA(Block), which adds the block branch of the MCA to the baseline network. (f) -w/ MCA(Grid), which adds the Grid branch of the MCA to the baseline network. (g) -w MSA-MCA, denotes our proposed texture sub-network. Table 3 and Fig 8 show the results of the ablation study. the results of MSA are able to highlight the foreground information of the image, but the image information is meshed. the colors of the results of MCA are not well balanced. the results of MSA-MCA are able to achieve pleasing visual effects. MSA-MCA has the highest PSNR and SSIM values from the quantitative results.

**Table 3:** Ablation studies of the texture sub-network.

Method	MSA		MCA		UIEB		SUID1	
	Block	Grid	Block	Grid	PSNR↑	SSIM↑	PSNR↑	SSIM↑
Baseline					20.7827	0.8152	14.9732	0.6784
-w/ MSA	✓	✓			24.3482	0.8994	16.283	0.7801
-w/ MSA(Block)	✓				24.2147	0.8975	16.2105	0.7747
-w/ MSA(Grid)		✓			23.9422	0.8976	15.6830	0.7631
-w/ MCA			✓	✓	24.2155	0.9009	16.1114	0.7822
-w/ MCA(Block)			✓		23.6807	0.8975	15.4049	0.7644
-w/ MCA(Grid)				✓	24.0706	0.9001	16.0042	0.7694
-w/ MSA-MCA	✓	✓	✓	✓	**24.5823**	**0.9012**	**16.9226**	**0.7930**

#### Effectiveness of the color sub-network

**Fig 9:**
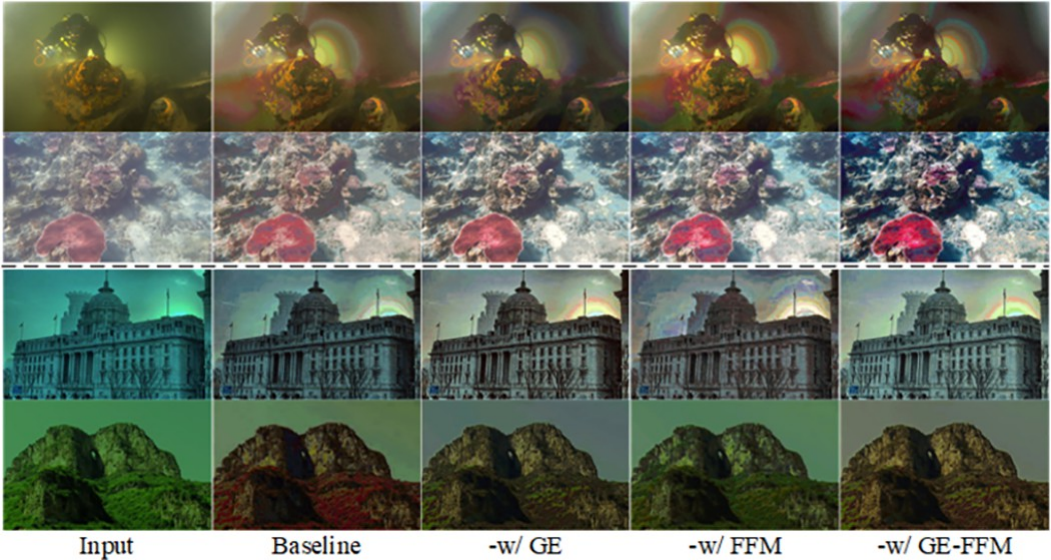
Qualitative ablation results for each key component of the color sub-network.

We use the texture subnetwork to extract image features as the baseline network, and we use colour reference loss for training. We add different components to the baseline network, i.e. (a) -w/ GE, which adds the GE module to the baseline network. (b) -w/ FFM, which adds the FFM module to the baseline network. (c) -w/ GE-FFM, which is our proposed color sub-network. Fig 9 and Table 4. show the results of the color sub-network ablation experiments. We can observe that: the colour sub-network is able to extract more rich color information; each module is able to achieve higher scores than the baseline to some extent; our proposed color network produces visually pleasing images but overemphasises local image colors.

**Table 4:** Ablation studies of the color sub-network.

Method	GE	FFM	UIEB		SUID1	
			PSNR↑	SSIM↑	PSNR↑	SSIM↑
Baseline			20.1767	0.8395	15.1776	0.6573
-w/ GE	✓		22.5395	0.8983	16.6988	0.7783
-W/ FFM		✓	22.2973	0.8723	16.1962	0.6969
-w/ GE-FFM	✓	✓	**23.8591**	**0.8980**	**16.8622**	**0.7909**

#### Effectiveness of the refinement network

**Fig 10:**
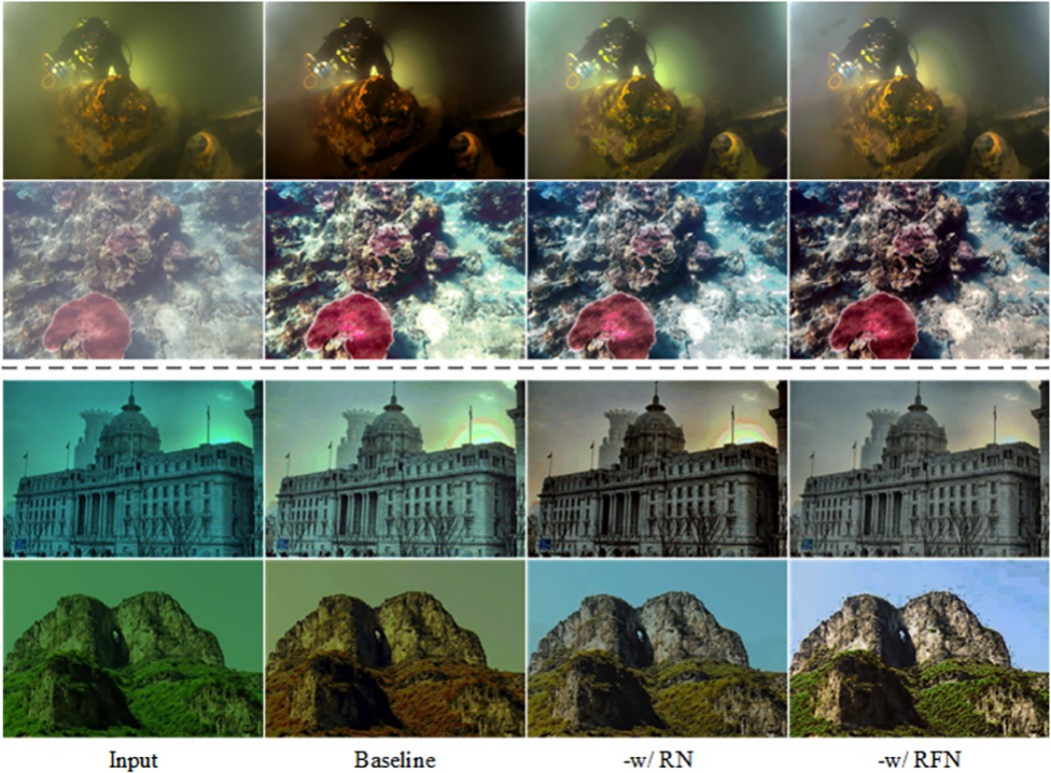
Qualitative ablation results for each key component of the refinement network.

We use the proposed simple connection (summation method) of the texture network and the colour network as the baseline and train with the total reference loss. We add different components to the baseline network, i.e., (a) -w/ RN, which adds the residual network to the baseline network. (b) -w/ RFN, Our proposed refinement network is added to the baseline network. Fig 10 and Table 5 show the results of the color ablation experiments.

**Table 5:** Ablation studies of the refinement network.

Method	RN	RFN	UIEB		SUID1	
			PSNR↑	SSIM↑	PSNR↑	SSIM↑
Baseline			24.8325	0.8939	17.0236	0.8007
-w/ RN	✓		25.6606	0.9164	17.2804	0.8470
-w/ RFN		✓	**25.7896**	**0.9234**	**17.4696**	**0.8927**

### Application tests

#### Structural enhancement

**Fig 11:**
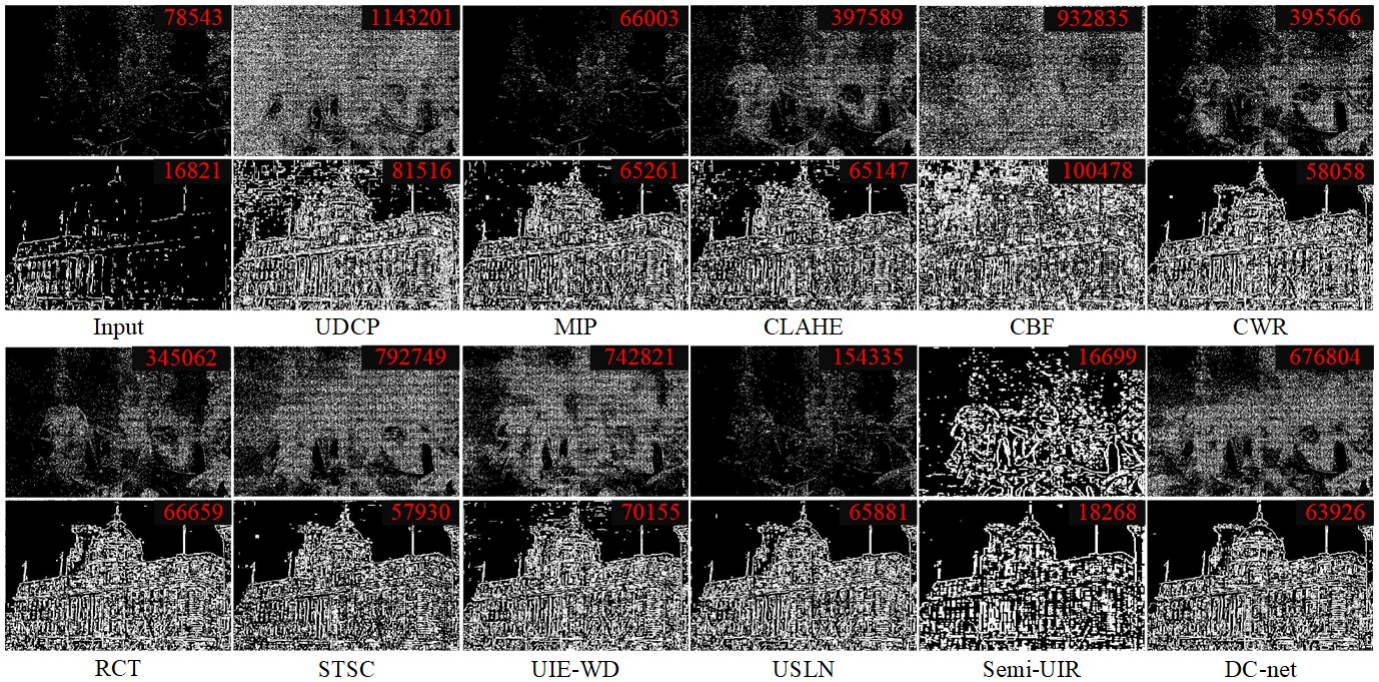
Comparison of the results of structural enhancement. The red numbers indicate the number of visible edges recovered by the algorithm, the first row shows the results of the real image treatment and the second row shows the results of the synthetic image protection.

To verify the structural enhancement superiority of the DC-net method, we use a blind contrast enhancement assessment method [47] to qualitatively and quantitatively evaluate the method. As shown in Fig 11, we can observe the following phenomena. 1) A high number of recovered edges is not a valid indication of the effectiveness of the enhancement algorithm, e.g. the UDCP and CBF methods are able to obtain a higher number of edges, but the noisy targets are also enhanced, leading to the objective confusion phenomenon. 2) The underwater image enhancement algorithm treats the synthetic image better than the real underwater image, showing that there are still some inherent differences between the synthetic underwater image and the real underwater image. 3) The structure of the underwater images handled by the advanced deep learning methods is more visible than the structure of the underwater images handled by the traditional learning methods. Among them, Semi-UIR was able to obtain the best results but yielded results with fewer target details to show. 4) The DC-net method is able to enhance the image target base and bring out more detail, e.g. the DC-net method shows a higher number of recovered edges than the Semi-UIR method.

#### Underwater image segmentation

**Fig 12:**
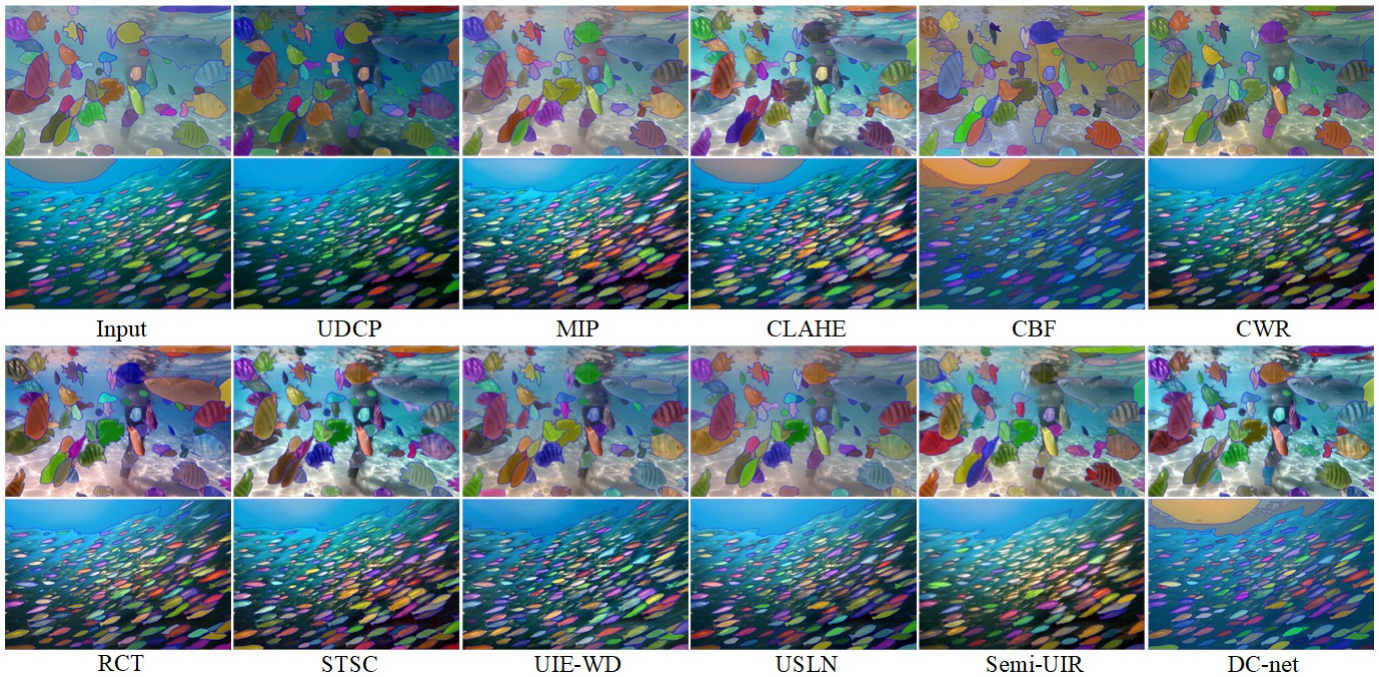
Underwater image segmentation results.

We use the segment anything algorithm [48] on the resulting image enhanced by all methods to detect as many targets as possible in the image. As shown in Fig 12, we can observe the following results 1) The results of different enhancement methods (low-level task) for underwater images do not show a positive correlation with image segmentation (high-level task). For example, the segmentation of conventional enhancement results (MIP, CLAHE) is not necessarily worse than the segmentation of deep enhancement results (USLN). 2) The image enhanced by DC-net proposed method is able to segment more targets with clear boundaries.

#### Underwater depth map estimation

**Fig 13:**
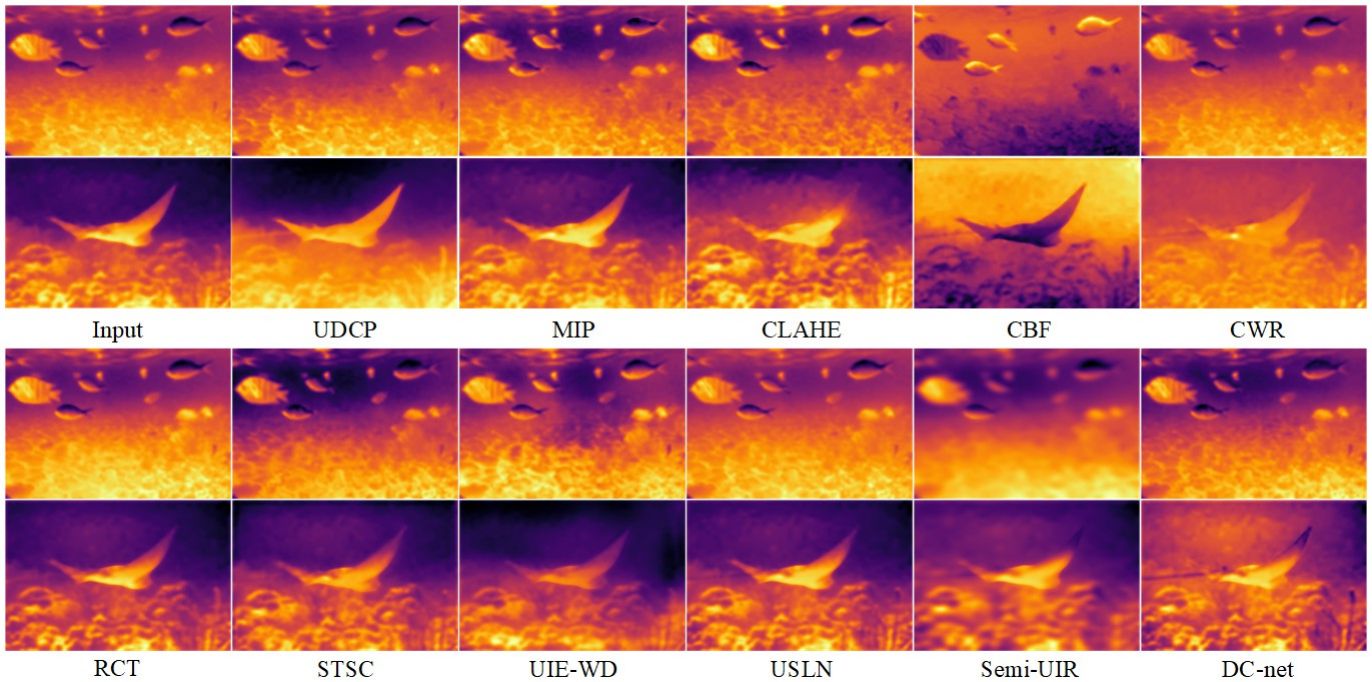
Underwater image depth estimation results.

We use a monocular depth estimation method [49] to handle the original underwater image and to enhance the depth map of the underwater image. As shown in Fig 13, the depth map of the image enhanced by the DC-net method contains finer and more accurate content than the enhanced images of other methods.

### Extension to image enhancement

**Fig 14:**
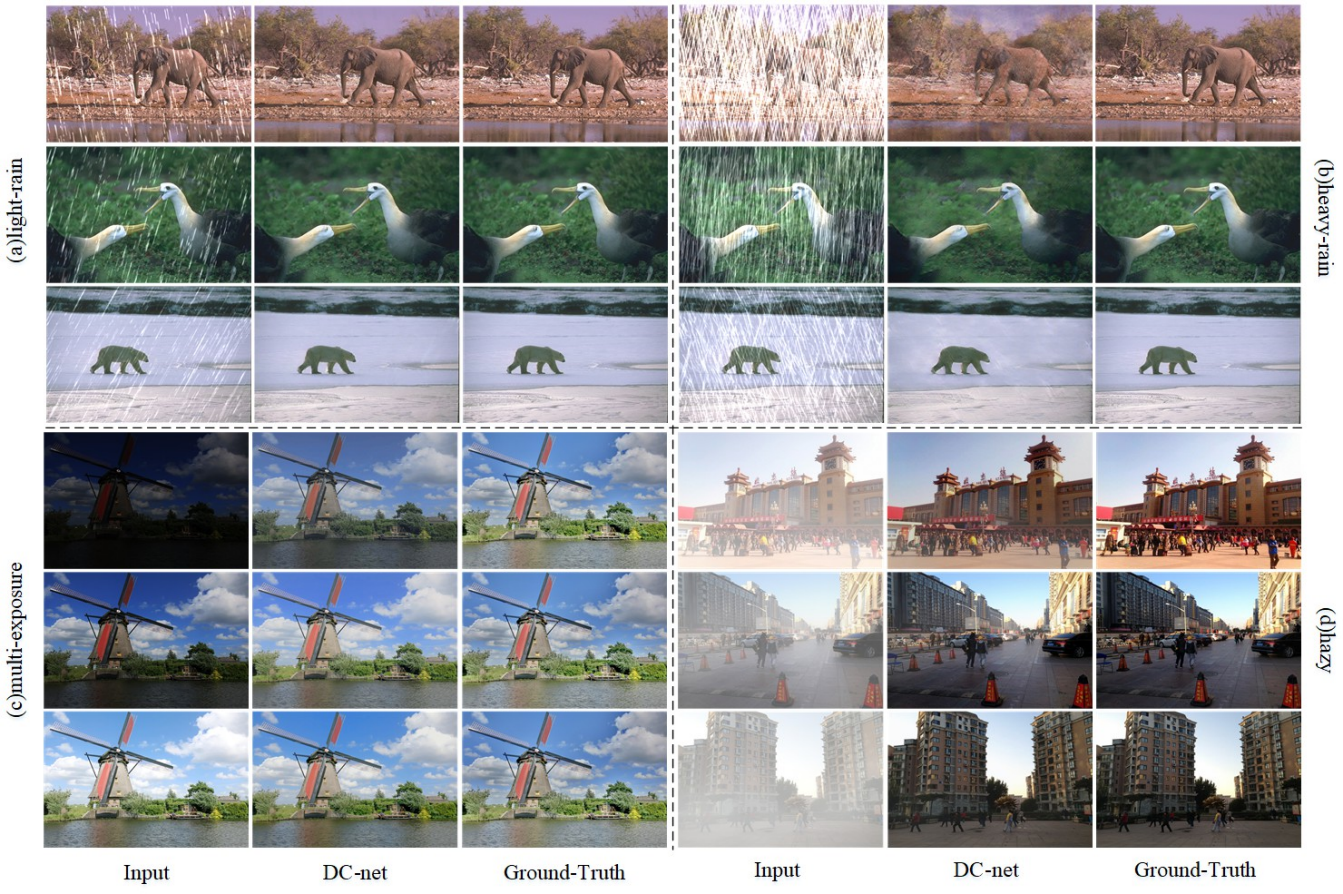
Experiment with weakly light, de-hazy and de-rain images.

The DC-net method proposed in this paper not only achieves better results in the field of underwater image enhancement but also makes an important breakthrough in the field of weakly light [50], de-hazy [51], and de-rain [52] images. As shown in Fig 14, the DC-net method is able to remove the effects of low light better in low visibility and contrast low light images. In images with different degrees of rain, we can process light rain images to achieve the expected results, but the recovery of heavy rain images is a bit worse, and traces of rain drops processing still appear in the recovered images. In images with fog, the DC-net method is able to obtain results with a natural appearance and clear details.

## Section 5: Conclusion

In this paper, we propose a divide-and-conquer network (DC-net) for underwater image distortion features based on the concept of decomposition feature learning. Specifically, in the texture network, to improve the extraction of texture feature information, we propose a multi-axis attention module to enhance the feature extraction capability of the network. In the color network, we design a LUTs module with fused features as input to achieve adaptive enhancement of color features. In addition, in order to reduce the possible negative impact of wide-range LUTs weights on underwater images, we propose a special gamma curve enhancement module, which can effectively mitigate its negative impact. Then, the enhanced results of the color and texture sub-networks are further integrated into the fusion module to obtain the enhanced results. Our proposed method is tested on a large number of underwater datasets to obtain state-of-the-art results.

Although we propose that DC-net networks can be effective in enhancing underwater images, there are however some limitations. First, as the method proposed in this paper performs feature fusion through a dual-stream network, it yields better results and also leads to the disadvantage of being computationally intensive. Second, many recent methods have shown that frequency domain methods can work in image enhancement, however, the method proposed in this paper only considers spatial domain image enhancement and does not consider recovering images from the frequency domain. Our future work will focus on addressing these issues.

## Supporting information

S1 Data (ZIP)
