# Supplementary material for: Underwater image enhancement using Divide-and-Conquer network
Source: PLoS One. 2024 Mar 5;19(3):e0294609. doi: 10.1371/journal.pone.0294609 (PMC10914272; doi:10.1371/journal.pone.0294609)
Supplement: S1 Data — (ZIP) [file pone.0294609.s001.zip › Supporting_Information/Appendix/appendix.pdf]

A Appendix

A.1 Comparison of six state-of-the-art methods

In order to further demonstrate the superiority of our approach, six additional state-of-the-art methods are tested here in a comparative experiment. Each approach is tested on a pre-trained model provided by the authors of the original article. Specific results are shown in Table A1 and Fig A1. The results show the superiority of our method, even if it does not achieve optimal results on some metrics, the combined results on the four experimental datasets show that our proposed method achieves superior results.

Table A1. Underwater image quality evaluation of different enhancement methods. The best results are marked in bold.

Dataset	Method	CycleGAN [1]	FUnIE-GAN [2]	LANET [3]	UGAN [4]	URESNE	USUIR [5]	DC-net
UIED	PSNR	22.4101	18.5975	18.9218	25.1386	24.1003	24.5069	**25.7896**
	SSIM	0.7484	0.7297	0.7688	0.9034	0.8834	0.8702	**0.9234**
	UCIQE	4.7663	5.6289	4.0869	5.0533	4.5853	4.9626	**5.8652**
	UIQM	0.7863	**1.2566**	0.6467	0.8885	0.9235	0.8875	1.1365
EUVP	PSNR	21.6668	19.1489	18.9929	20.6157	21.4037	21.2527	**22.8644**
	SSIM	0.6815	0.6550	0.7336	0.7329	0.7175	0.7158	**0.7686**
	UCIQE	4.4849	**5.3558**	4.3623	4.6157	4.3798	4.8262	4.0983
	UIQM	0.6582	**0.9297**	0.7014	0.7552	0.7761	0.7783	0.8799
SUID	PSNR	15.2069	14.7409	20.5668	15.2474	17.2345	17.3842	**23.5689**
	SSIM	0.6247	0.5835	0.7849	0.7139	0.6861	0.6998	**0.8924**
	UCIQE	3.2838	4.4033	2.8133	3.6953	3.9162	4.5732	**5.8142**
	UIQM	0.7062	0.9757	0.5007	0.8393	0.8184	0.7312	**1.0562**
SUID1	PSNR	20.4587	14.9713	15.6111	21.3761	17.3974	**22.6445**	17.4696
	SSIM	0.7356	0.6700	0.7652	0.8421	0.7881	0.8392	**0.8927**
	UCIQE	4.2059	4.0569	3.0221	4.1301	4.0063	**5.1897**	4.9095
	UIQM	0.9871	**1.1559**	0.4859	1.0657	1.0856	1.0288	0.7316

Fig A1. Visualization of the comparision results.

A.2 Complementary experiments on ablation of texture networks.

We first use the unet-like network as our baseline network and train it using texture reference loss. We add different components to the baseline network, i.e. (a) -w/

SA-CA, adding the SA-CA module to the baseline network. (b) -w/ MSA-CA, adding the MSA-CA module to the baseline network. (c) -w/ SA-MCA, adding the SA-MCA module to the baseline network. (d) -w/ MSA-MCA, adding the MSA-MCA module to the baseline network. The specific experimental results are shown in Table A3 and Fig A2, where we can observe the following conclusions.

a) We can observe that when we only use common spatial-channel attention, it will lead to satisfactory outputs (+1.4618dB). This phenomenon suggests that attention mechanisms can be effective in improving the quality of underwater images.

b) Note that the huge jump of PSNR by adding multi-axis on channel and spatial attention respectively, which can be largely attributed to the addition of local and global information layers. -w/ MSA-CA shows the PSNR is greatly improved, -w/ SA-MC shows the SSIM is greatly improved.

c) Overall, we observe a huge improvement when including -w/ MSA-MCA, because it incorporates both local and global information on the channel and spatial.

Table A2. Ablation studies of the multi-axis attention. "SA" stands for Common Spatial attention (Spatial Attention Mechanisms, SAM [6]). "CA" stands for Common Channel attention (Squeeze-and-Excitation Networks, SEnet [7]). "MSA" stands for Multi-axis Spatial attention. "MCA" stands for Multi-axis Channel attention.

Method	SA	CA	MSA	MCA	PSNR↑	SSIM↑
Baseline					20.7827	0.8152
-w/ SA-CA	✓	✓			22.2445	0.8395
-w/ MSA-CA		✓	✓		23.6261	0.8659
-w/ SA-MCA	✓			✓	23.2747	0.8980
-w/ MSA-MCA			✓	✓	**24.5823**	**0.9012**

Fig A2. Qualitative ablation results for each key component of our method on the UIEB datasets.

We construct separate multi-axis attention modules (block attention and grid attention) on the spatial and channel axes, but how can the two be combined to achieve optimal results? We investigate six permutations of both spatial axis attention and channel axis attention, and the results of the scores are shown in Table A3. We find that a parallel combination work best: MSA(p)-MCA(p). The reason may be that the parallel designs learn complementary cues, which interact less with each other to be able to maintain more features.

A.3 Param and Flops comparisons.

In this subsection we have tested several methods to compare the number of operations and parameters and the results are shown in Table A4. We use 256*256 inputs to measure the parameters associated with each algorithm. From the experimental results, we can see that our method is the most computationally intensive method.

Table A4. Comparison of parameter counts, floating-point operation per second.

Method	CWR	STSC	Semi-UIR	USLN	UIE-WD	DC-net
Flops [G]	123.68	2426.34	364.38	0.56	513.76	1865.83
Param [M]	5.4863	69.2950	1.5769	0.0085	13.0694	63.6324

Table A3. Multi-axis attention block order study. "MSA" stands for Multi-axis Spatial Attention. "MCA" stands for Multi-axis Channel Attention. "(s)" stands for the sequential stacking of block attention and grid attention, and "(p)" represents the parallel processing of block attention and grid attention.

Method	PSNR↑	SSIM↑
MSA(s)-MCA(p)	23.5648	0.8726
MCA(s)-MSA(p)	23.7896	0.8836
MSA(p)-MCA(s)	23.5687	0.7689
MCA(p)-MSA(s)	22.3698	0.8245
MCA(s)-MSA(s)	23.9863	0.8926
MSA(p)-MCA(p)	**24.5823**	**0.9012**

References

1. Jun-Yan Zhu, Taesung Park, Phillip Isola, and Alexei A Efros. Unpaired image-to-image translation using cycle-consistent adversarial networks. In *Proceedings of the IEEE international conference on computer vision*, pages 2223–2232, 2017.

2. Md Jahidul Islam, Youya Xia, and Junaed Sattar. Fast underwater image enhancement for improved visual perception. *IEEE Robotics and Automation Letters*, 5(2):3227–3234, 2020.

3. Hanning Yu, Wentao Liu, Chengjiang Long, Bo Dong, Qin Zou, and Chunxia Xiao. Luminance attentive networks for hdr image and panorama reconstruction. In *Computer Graphics Forum*, volume 40, pages 181–192. Wiley Online Library, 2021.

4. Cameron Fabbri, Md Jahidul Islam, and Junaed Sattar. Enhancing underwater imagery using generative adversarial networks. In *2018 IEEE international conference on robotics and automation (ICRA)*, pages 7159–7165. IEEE, 2018.

5. Zhenqi Fu, Huangxing Lin, Yan Yang, Shu Chai, Liyan Sun, Yue Huang, and Xinghao Ding. Unsupervised underwater image restoration: From a homology perspective. In *Proceedings of the AAAI Conference on Artificial Intelligence*, volume 36, pages 643–651, 2022.

6. Xizhou Zhu, Dazhi Cheng, Zheng Zhang, Stephen Lin, and Jifeng Dai. An empirical study of spatial attention mechanisms in deep networks. In *Proceedings of the IEEE/CVF international conference on computer vision*, pages 6688–6697, 2019.

7. Jie Hu, Li Shen, and Gang Sun. Squeeze-and-excitation networks. In *Proceedings of the IEEE conference on computer vision and pattern recognition*, pages 7132–7141, 2018.
