# Supplementary figures and images for: Underwater image enhancement using Divide-and-Conquer network
Source: PLoS One. 2024 Mar 5;19(3):e0294609. doi: 10.1371/journal.pone.0294609 (PMC10914272; doi:10.1371/journal.pone.0294609)

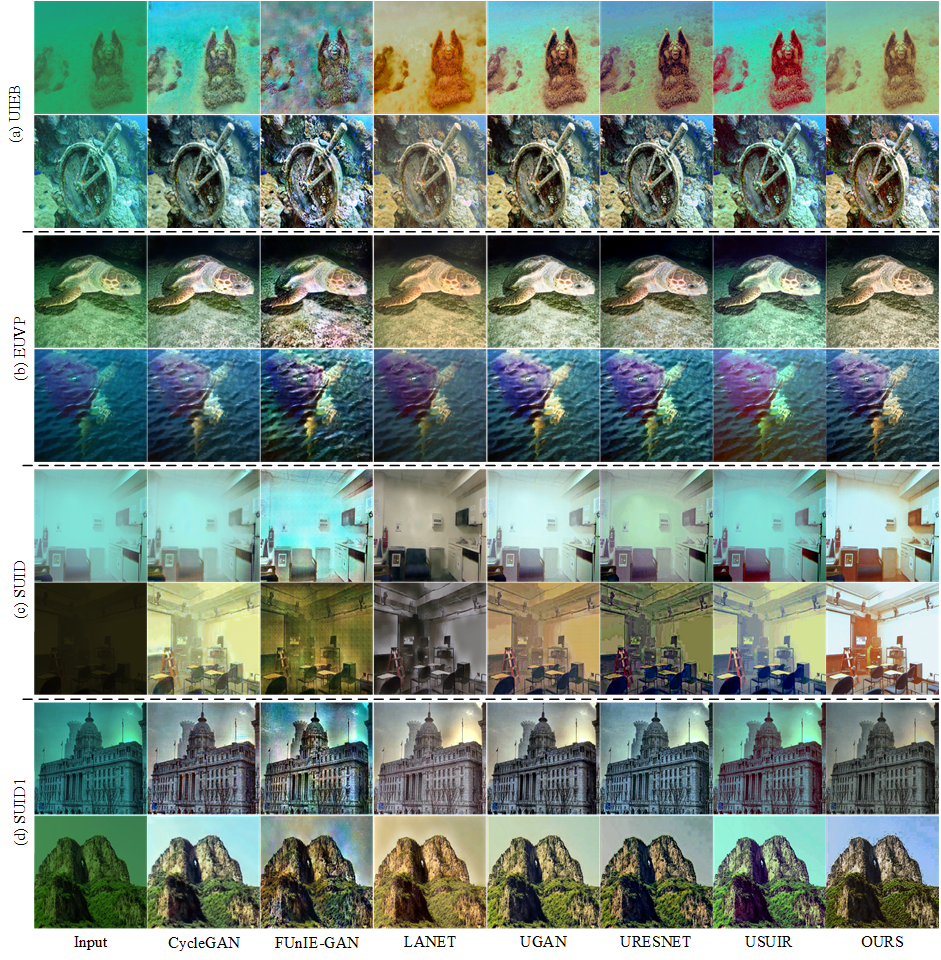

Supplement: S1 Data — (ZIP) [file pone.0294609.s001.zip › Supporting_Information/Appendix/figA1.tif]

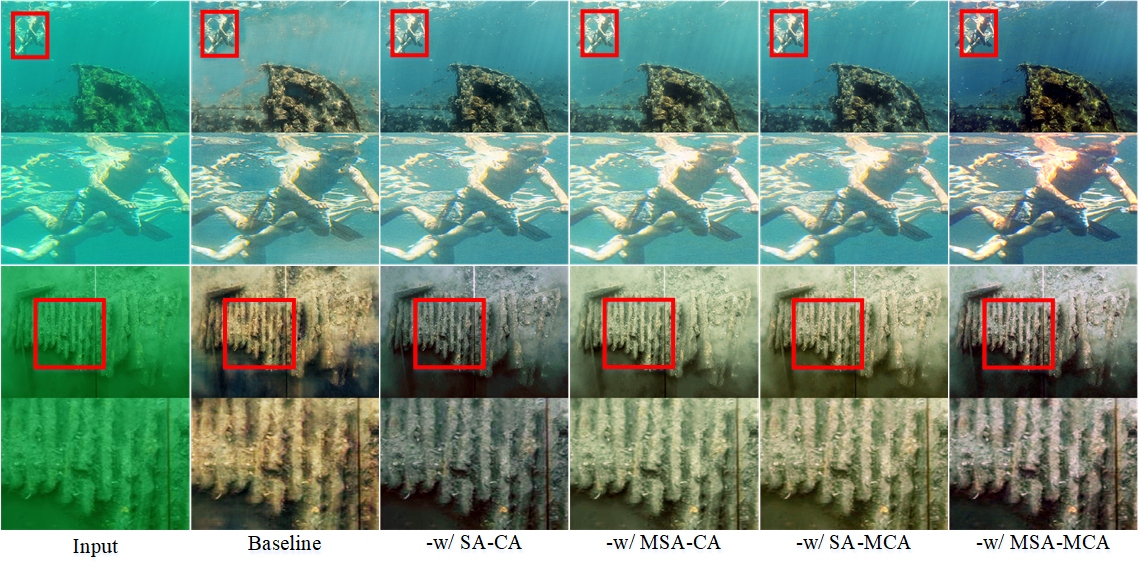

Supplement: S1 Data — (ZIP) [file pone.0294609.s001.zip › Supporting_Information/Appendix/figA2.tif]

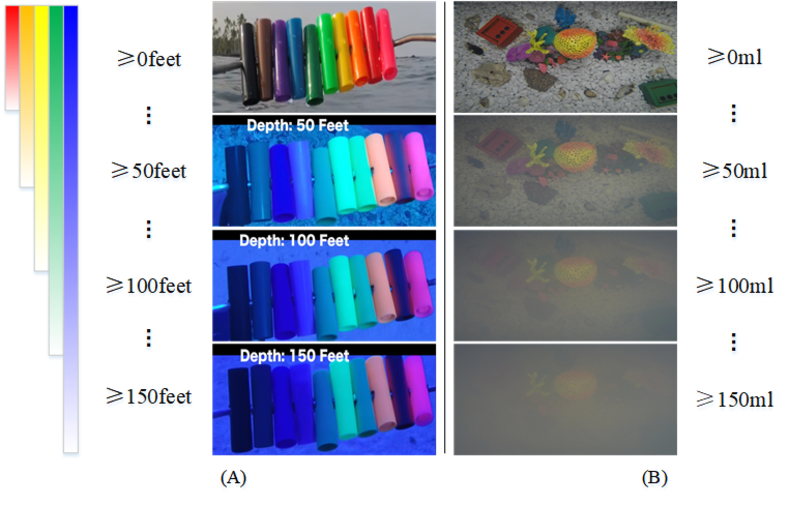

Supplement: S1 Data — (ZIP) [file pone.0294609.s001.zip › Supporting_Information/manuscript_figure/fig1.tif]

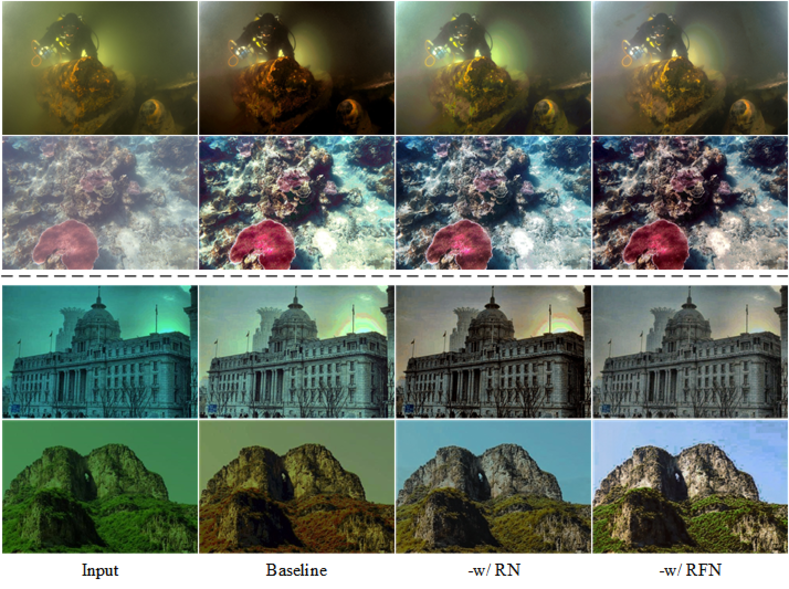

Supplement: S1 Data — (ZIP) [file pone.0294609.s001.zip › Supporting_Information/manuscript_figure/fig10.tif]

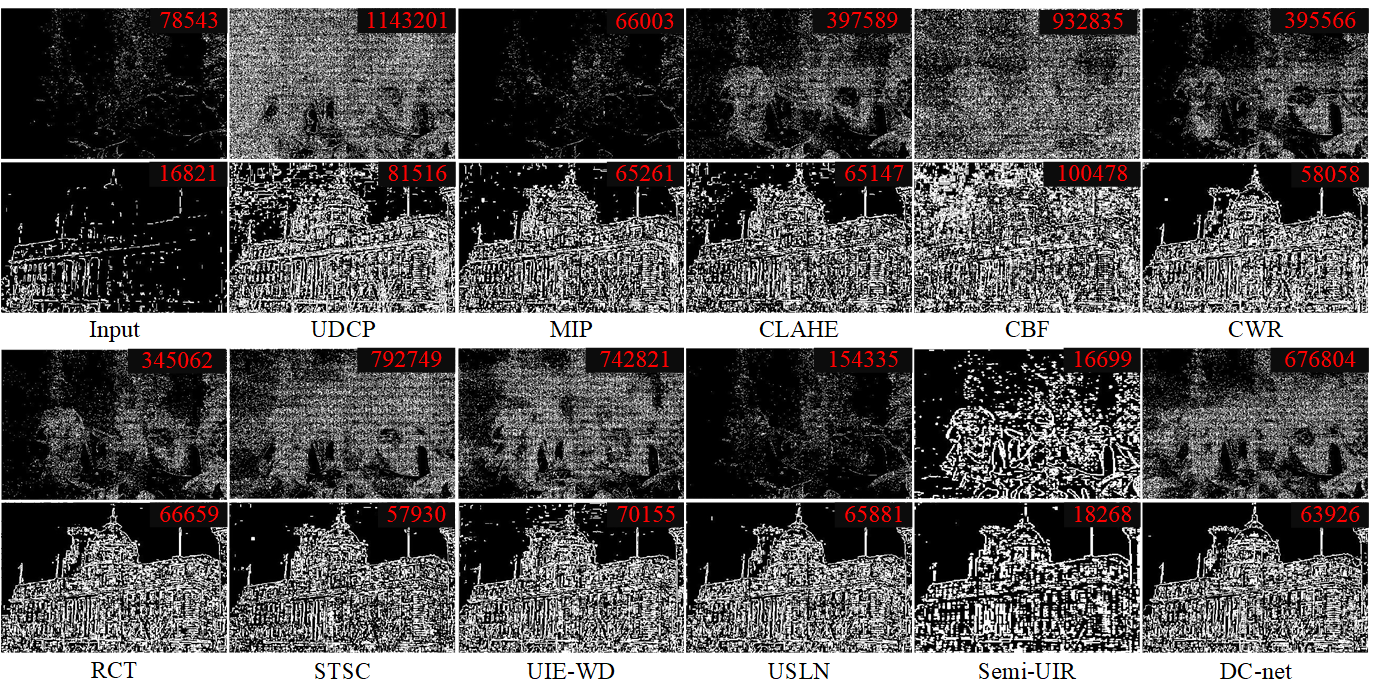

Supplement: S1 Data — (ZIP) [file pone.0294609.s001.zip › Supporting_Information/manuscript_figure/fig11.tif]

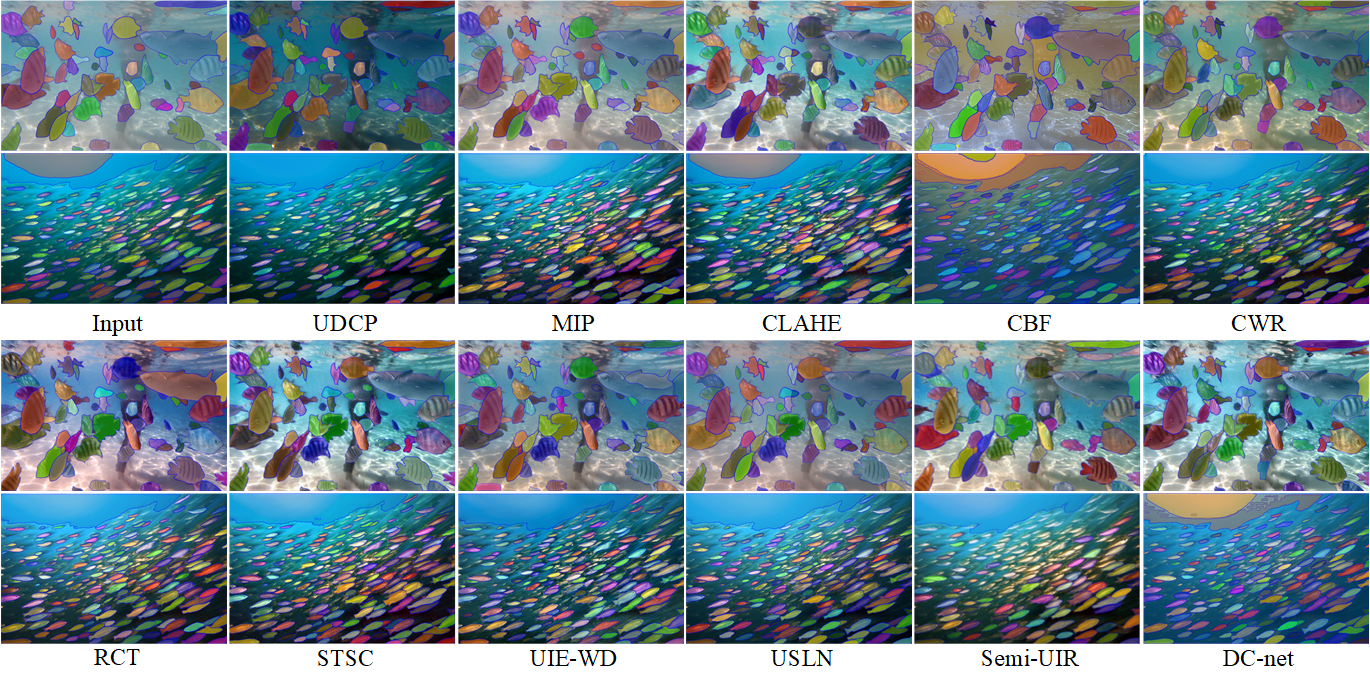

Supplement: S1 Data — (ZIP) [file pone.0294609.s001.zip › Supporting_Information/manuscript_figure/fig12.tif]

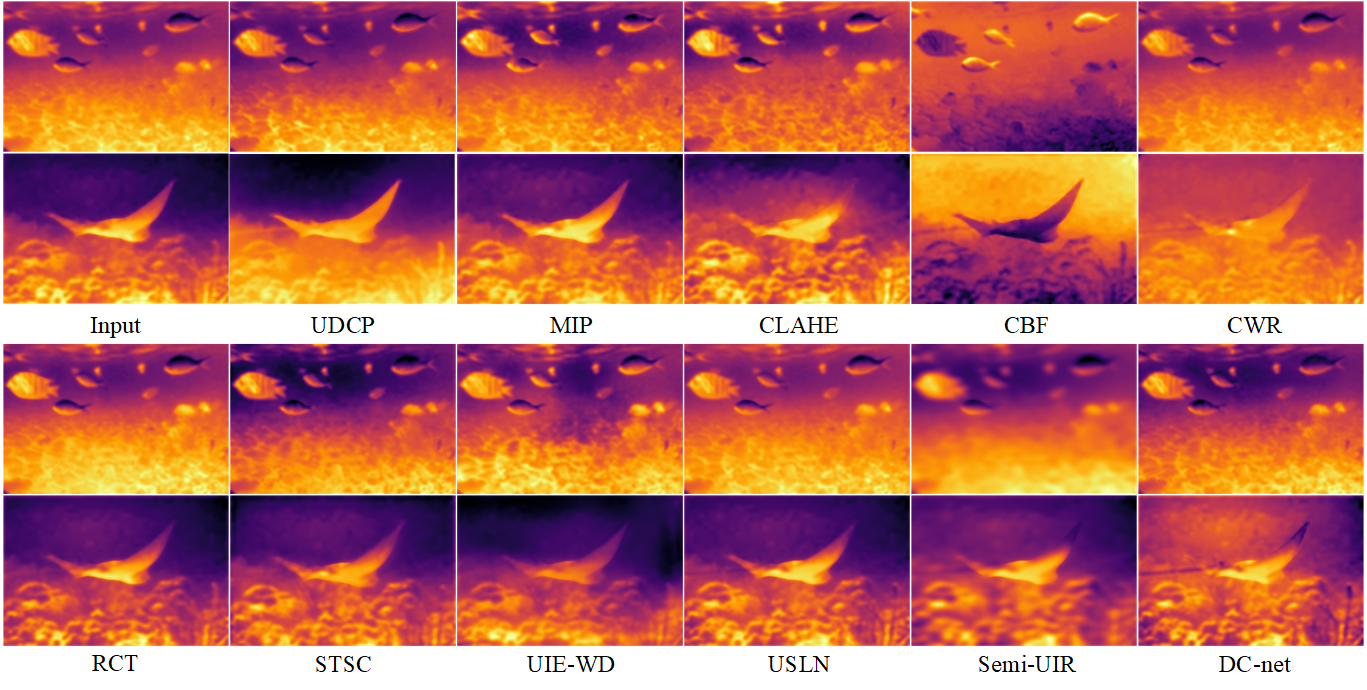

Supplement: S1 Data — (ZIP) [file pone.0294609.s001.zip › Supporting_Information/manuscript_figure/fig13.tif]

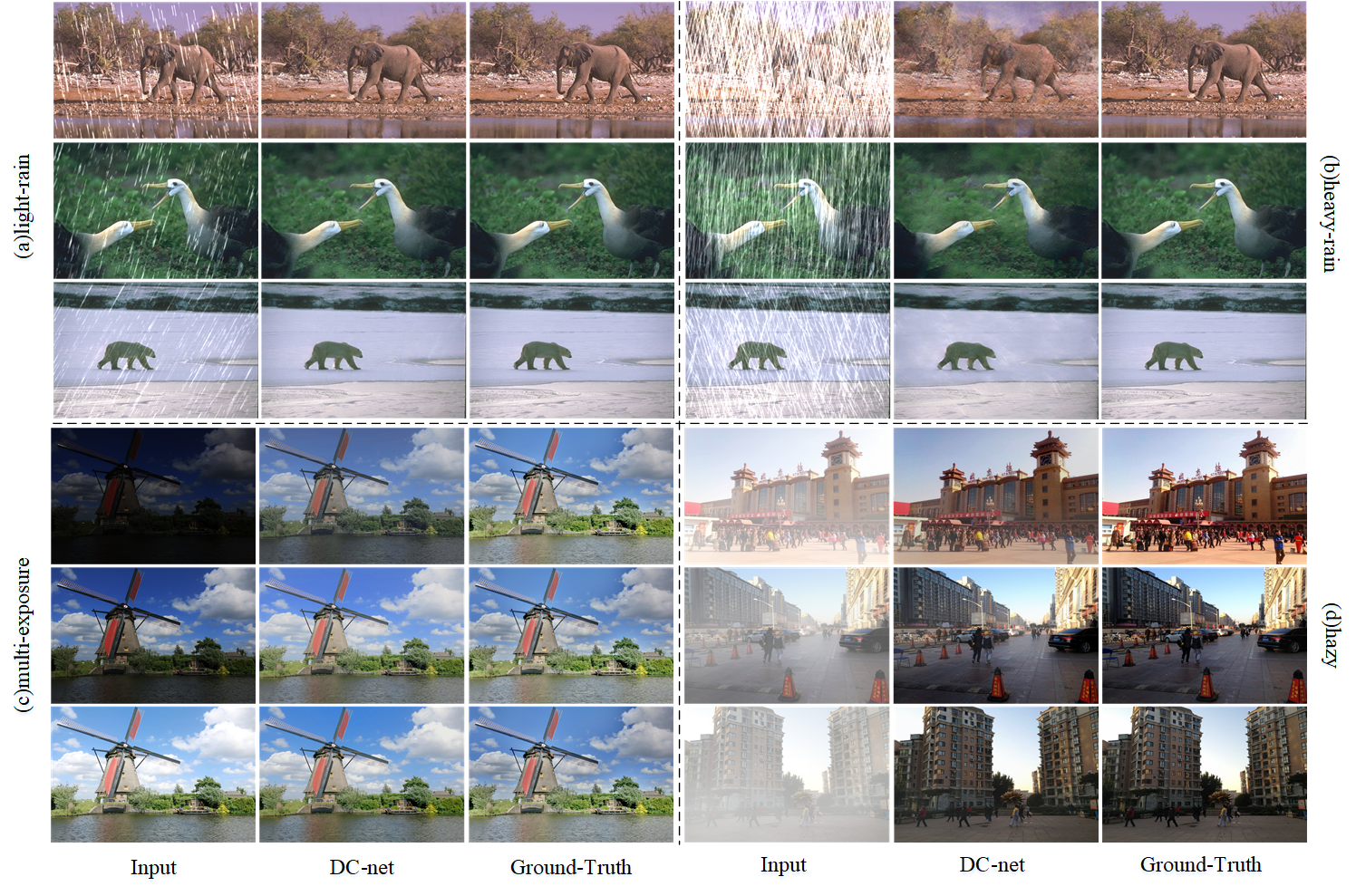

Supplement: S1 Data — (ZIP) [file pone.0294609.s001.zip › Supporting_Information/manuscript_figure/fig14.tif]

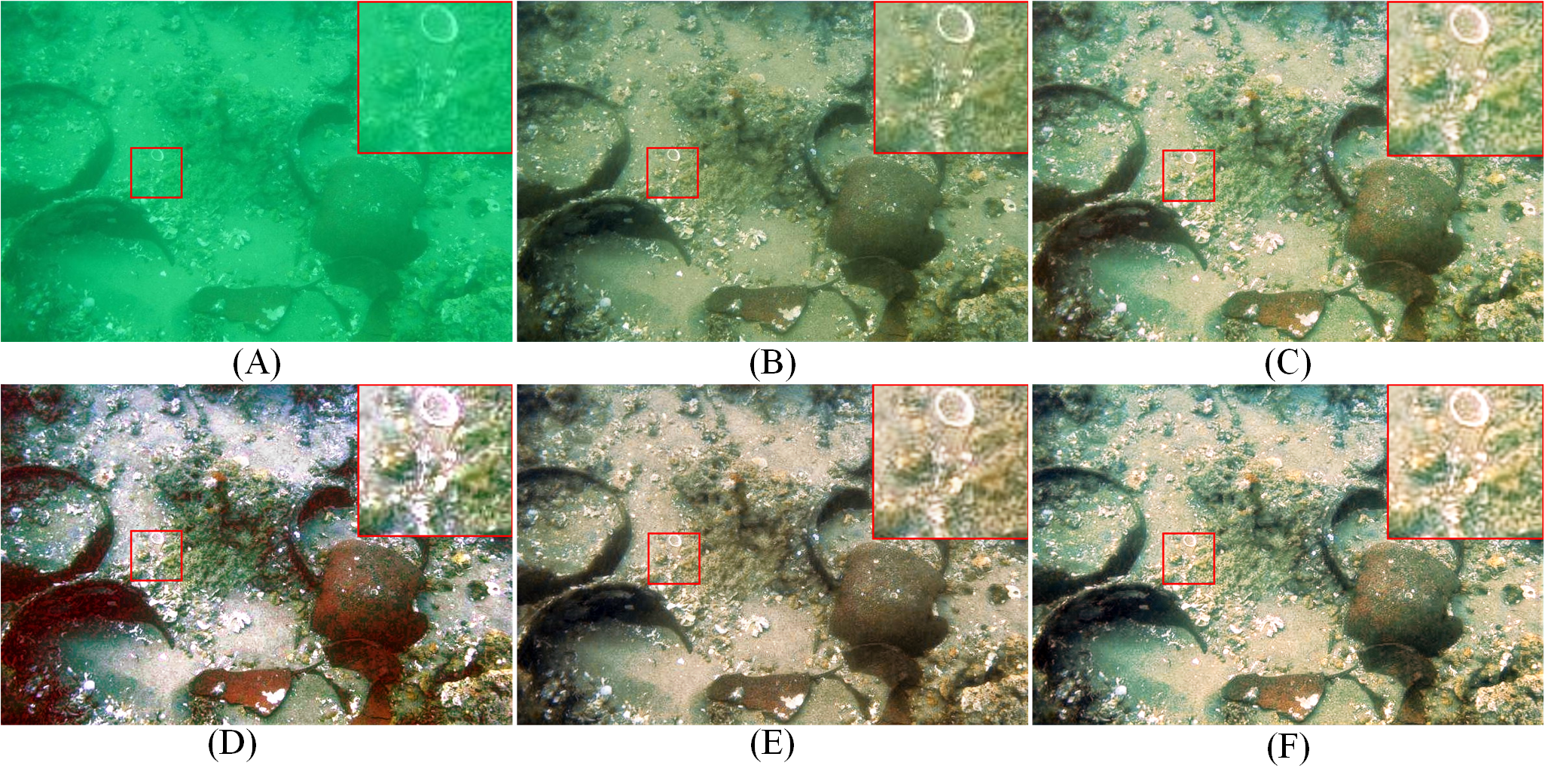

Supplement: S1 Data — (ZIP) [file pone.0294609.s001.zip › Supporting_Information/manuscript_figure/fig2.tif]

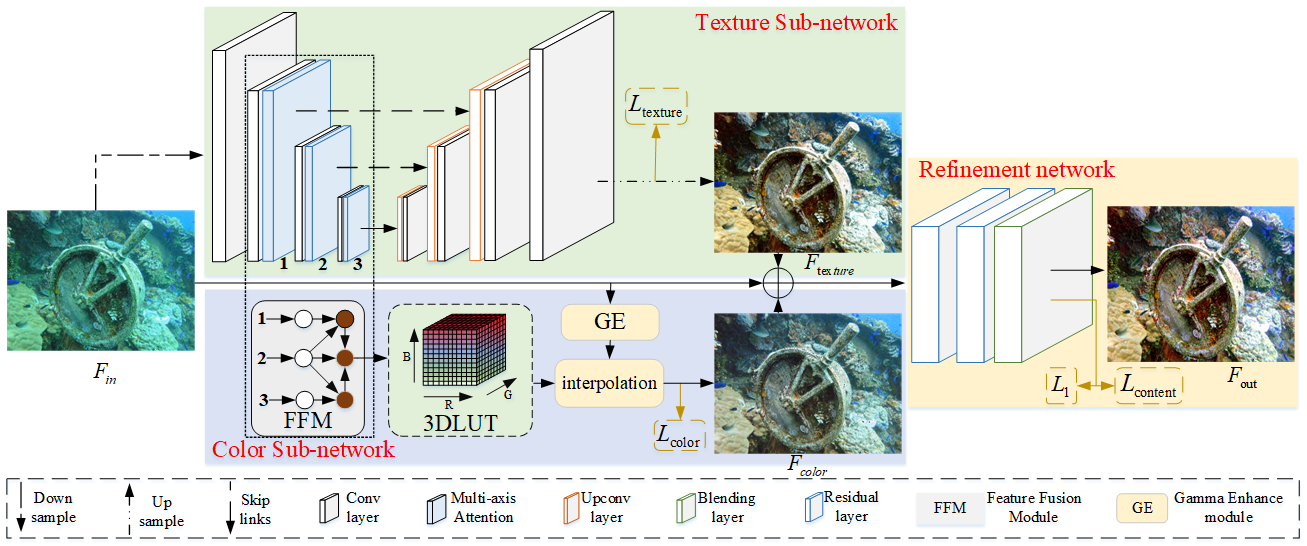

Supplement: S1 Data — (ZIP) [file pone.0294609.s001.zip › Supporting_Information/manuscript_figure/fig3.tif]

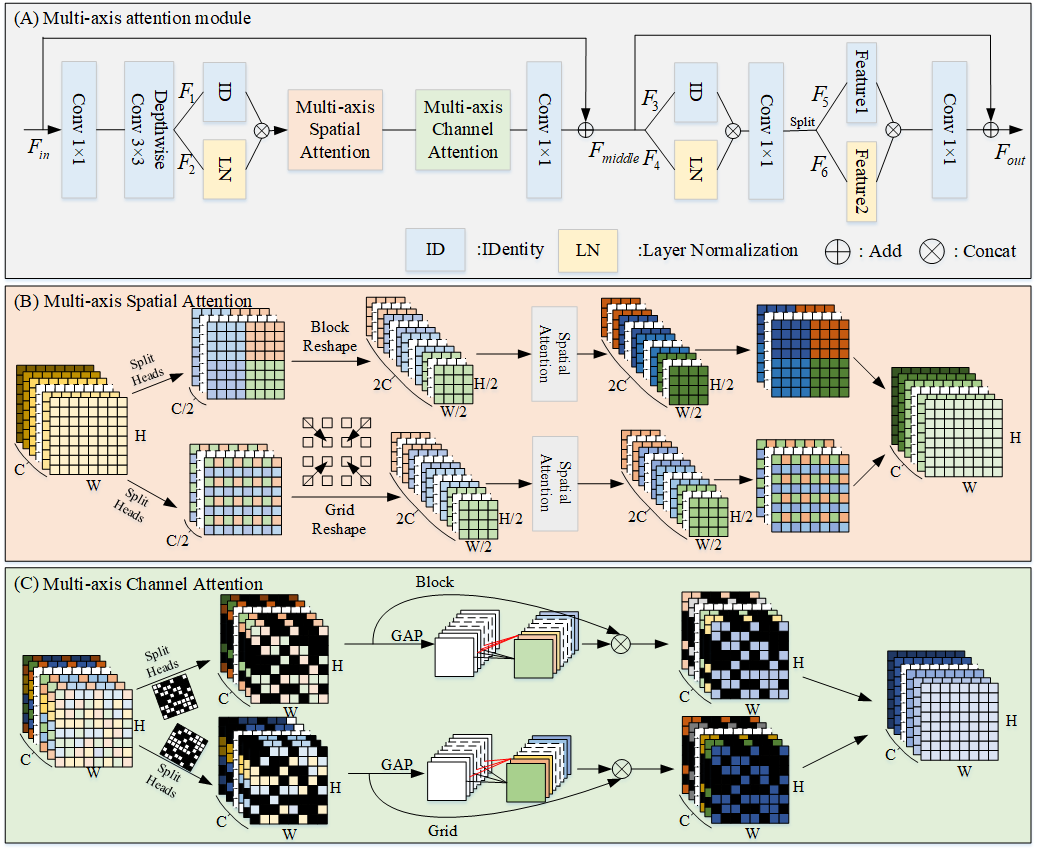

Supplement: S1 Data — (ZIP) [file pone.0294609.s001.zip › Supporting_Information/manuscript_figure/fig4.tif]

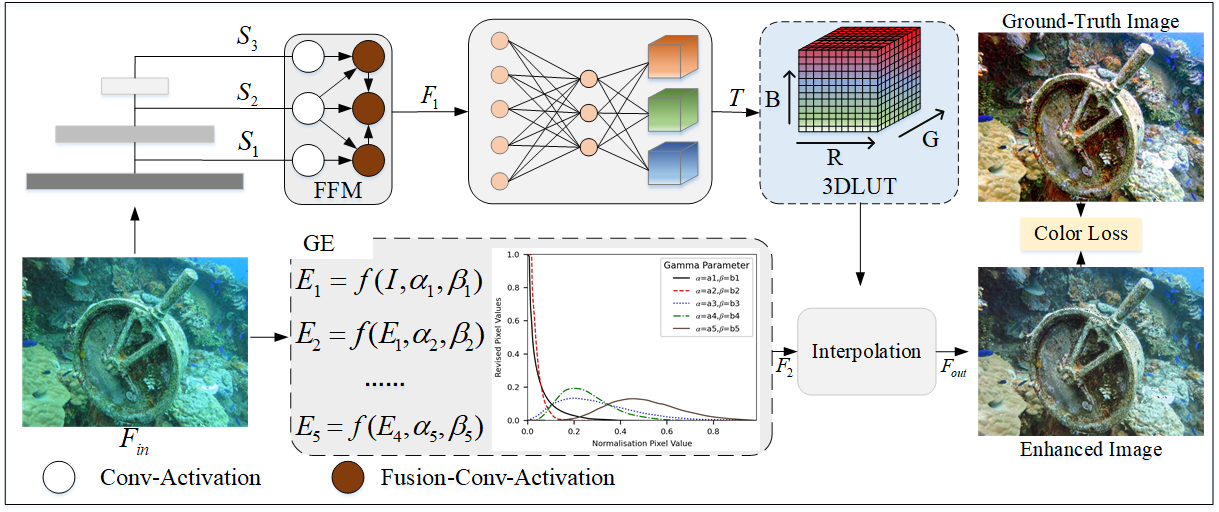

Supplement: S1 Data — (ZIP) [file pone.0294609.s001.zip › Supporting_Information/manuscript_figure/fig5.tif]

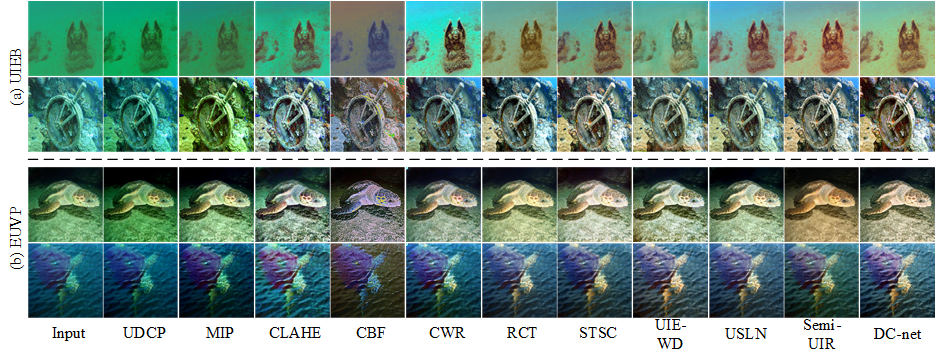

Supplement: S1 Data — (ZIP) [file pone.0294609.s001.zip › Supporting_Information/manuscript_figure/fig6.tif]

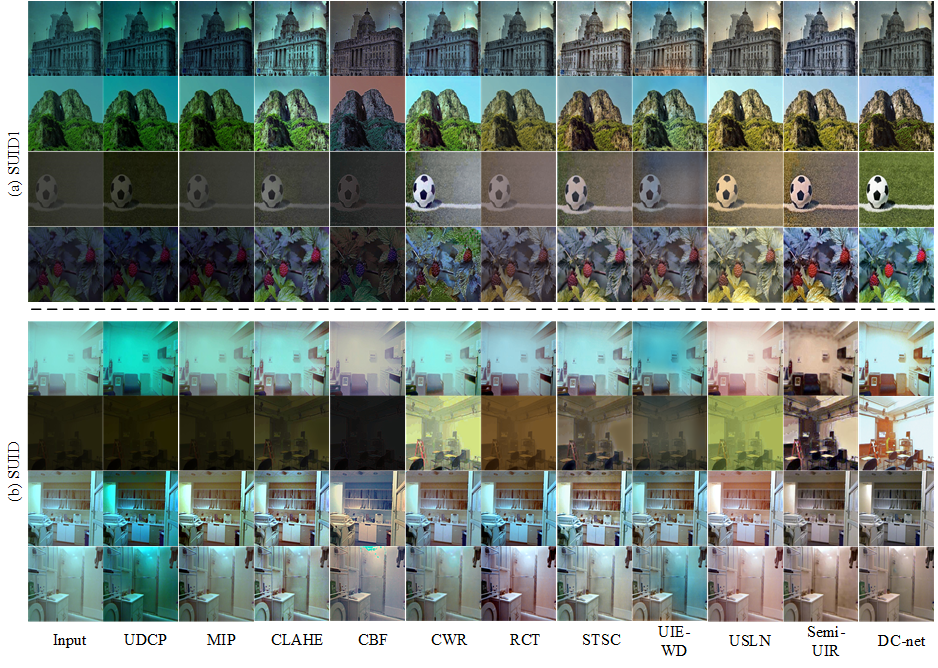

Supplement: S1 Data — (ZIP) [file pone.0294609.s001.zip › Supporting_Information/manuscript_figure/fig7.tif]

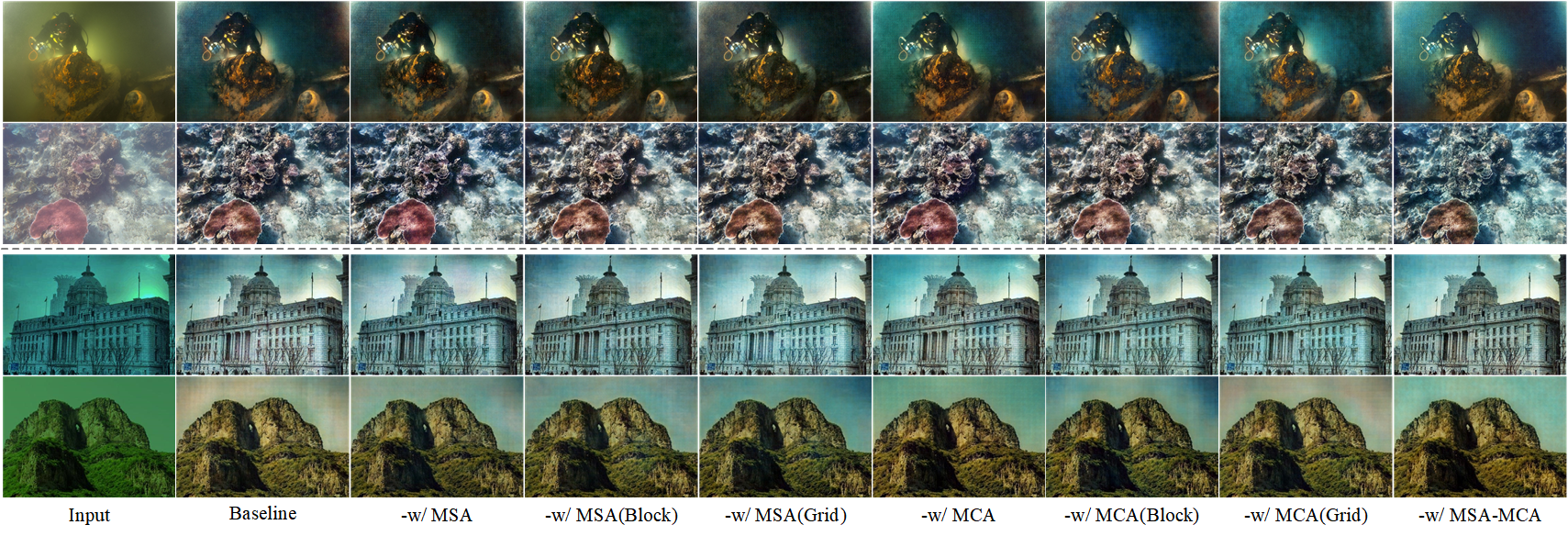

Supplement: S1 Data — (ZIP) [file pone.0294609.s001.zip › Supporting_Information/manuscript_figure/fig8.tif]

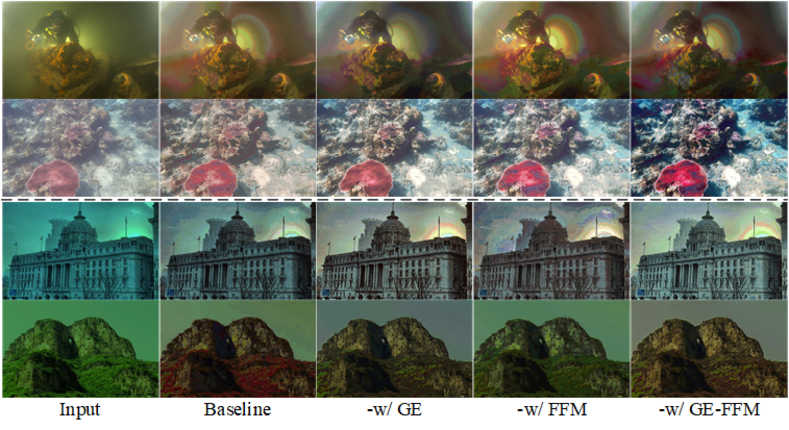

Supplement: S1 Data — (ZIP) [file pone.0294609.s001.zip › Supporting_Information/manuscript_figure/fig9.tif]
